# ﻿*Lysathiaflavipes* and *Lysathiacilliersae* Cabrera sp. nov. (Coleoptera, Chrysomelidae): genetic and morphological unravelling of biocontrol agents for two invasive aquatic plants

**DOI:** 10.3897/zookeys.1228.138773

**Published:** 2025-02-13

**Authors:** Ana C. Faltlhauser, Nora Cabrera, M. Cristina Hernández, Andrés F. Sánchez Restrepo, Martin Hill, Alejandro J. Sosa

**Affiliations:** 1 Fundación para el Estudio de Especies Invasivas (FuEDEI), Hurlingham, Argentina Fundación para el Estudio de Especies Invasivas Hurlingham Argentina; 2 Consejo Nacional de Investigaciones Científicas y Técnicas, CABA, Argentina Consejo Nacional de Investigaciones Científicas y Técnicas CABA Argentina; 3 Facultad de Cs. Naturales y Museo, Universidad de La Plata, La Plata, Argentina Universidad de La Plata La Plata Argentina; 4 Department of Zoology and Entomology, Centre for Biological Control (CBC), Rhodes University, Makhanda, South Africa Rhodes University Makhanda South Africa

**Keywords:** Alticini, biological control, DNA barcoding, haplotype, integrative taxonomy, Parrot’s feather, Water primrose

## Abstract

In the search for specific natural enemies to control two invasive aquatic plants (IAP) from South America, Ludwigiagrandiflorasubsp.hexapetala (Onagraceae) and *Myriophyllumaquaticum* (Haloragaceae), taxonomic challenges associated with two *Lysathia* Bechyné, 1959 (Chrysomelidae; Alticini) species had to be resolved. *Lysathiaflavipes* (Boheman, 1859) exhibits significant morphological variation, causes heavy damage to both IAPs, and may represent more than one species due to the phylogenetic gap between hosts. Additionally, an undescribed *Lysathia* species (previously published as *Lysathia* sp.), sourced from Brazil, has been successfully used as a control agent for *M.aquaticum* in South Africa since 1994. An integrative taxonomic approach combining genetic and morphological analyses was employed. A lectotype and paralectotypes for *Graptoderaflavipes* Boheman, 1859 are here designated. Phylogenetic studies revealed that *L.flavipes* had greater genetic and morphological variation than originally described, and no evidence suggested that *L.flavipes* represented a species complex associated with its host plants. As a result, the species description was expanded. On the other hand, genetic and morphological differences such as body size, colouration, and genital structures further supported the description of *Lysathiacilliersae* Cabrera, **sp. nov.** and its differentiation from other closely related species, including *L.flavipes* and *L.ludoviciana* (Fall, 1910). Specimens of *L.cilliersae***sp. nov.** collected in Misiones, Argentina, matched those from South Africa. Genetic sequences correlated with morphological vouchers, images, and illustrations of morphology and genitalia, as well as new distribution records, are provided. This research contributes to the taxonomic knowledge of the *Lysathia* genus and supports accurate species identification in applied entomological contexts, such as biological control programmes.

## ﻿Introduction

Invasive aquatic plants (IAP) have both ecological and economic impacts that threaten ecosystems (Hussner et al. 2017). Reduction of biodiversity and water quality, disruption of ecosystems by outcompeting native species, and altering habitats are some of the various challenges that these types of species pose (Stiers et al. 2011; Thouvenot et al. 2013). Traditional approaches to managing IAP often involve mechanical and chemical methods (Andreu et al. 2009); however, they can have limitations such as non-specificity, short-term effectiveness, and environmental concerns (Hussner et al. 2017). Given these challenges, biological control, which involves introducing monophagous natural enemies (insects, pathogens, or herbivores) to manage invasive species by reducing their population sizes or limiting their spread (Cruttwell McFadyen 1998), has gained attention as a control method (Sankaran et al. 2023). This approach offers several advantages, including long-term effectiveness, reduced environmental impact compared to chemical methods, and potential for self-sustaining control (Culliney 2005; McFadyen 2008; Hinz et al. 2020).

**Figure 1.**
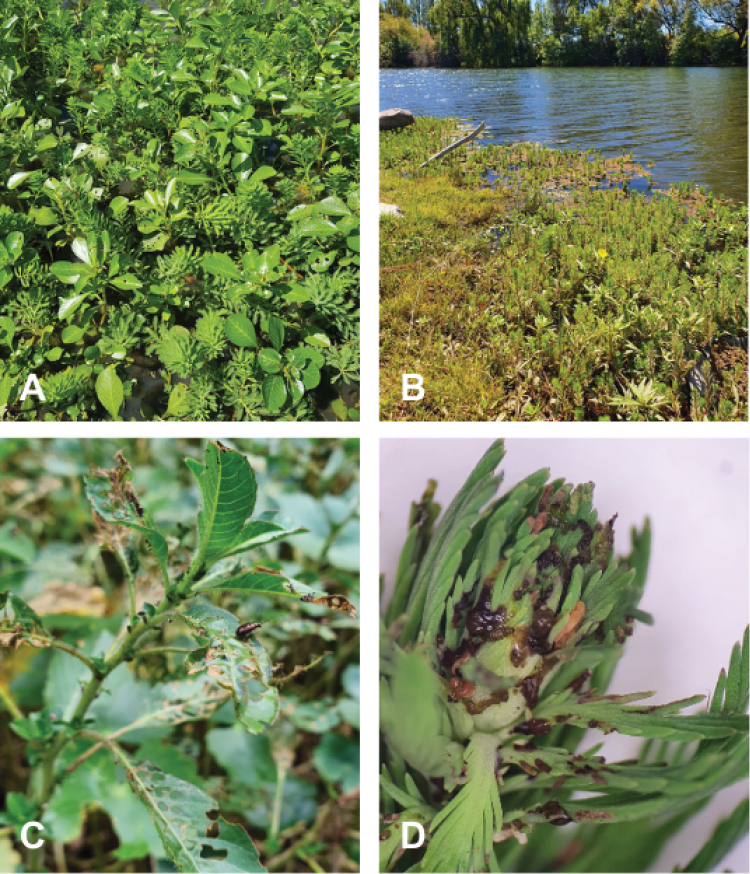
Ludwigiagrandiflorasubsp.hexapetala and *Myriophyllumaquaticum* coexisting in the same environment in Argentina, localities of **A** Calabacillas (Entre Ríos) and **B** Plottier (Neuquén) **C** Damage of *Lysathiaflavipes* on L.g.subsp.hexapetala**D***L.flavipes* larval damage on *M.aquaticum*.

Water primrose (Ludwigiagrandiflorasubsp.hexapetala (Hook. & Arn.) G.L. Nesom & Kartesz; Onagraceae) and parrot’s feather (*Myriophyllumaquaticum* (Vell.) Verdc.; Haloragaceae) are aquatic plants native to South America (Raven and Axelrod 1974; Orchard 1981; Sutton 1985; Sytsma et al. 2004; Wagner et al. 2007). In its native range in Argentina, they typically coexist in their natural environment (Fig. 1A, B), growing in shallow waters across the centre and north of the country, reaching northern Patagonia (Sabbatini et al. 1998). Conversely, they are considered aggressive IAPs in North America (Reddy et al. 2021b), Europe (Di Pietro et al. 2007; Thouvenot et al. 2013), Africa (Hill and Coetzee 2017), and Oceania (Hussner et al. 2017), where they obstruct water flow and cause various environmental and economic problems by forming dense mats that cover water bodies, streams, and irrigation channels (Pelella et al. 2023; Rojas-Sandoval 2024). Chemical or mechanical strategies against L.g.subsp.hexapetala and *M.aquaticum* have proven to be inefficient. For this reason, several projects are either developing integrated management approaches that include biological control or have already implemented biological control programmes against these weeds (Cilliers 1999a; Sheppard et al. 2006; Oberholzer et al. 2007; Scott et al. 2020; Reddy et al. 2021b; Lesieur et al. 2023; Pessina et al. 2024).

In the search of specific natural enemies for L.g.subsp.hexapetala and *M.aquaticum*, there is a particular interest to solve taxonomic challenges associated with two flea beetle species (Coleoptera: Chrysomelidae: Galerucinae: Alticini) within the genus *Lysathia* Bechyné, 1959. Surveys in Argentina have revealed frequent and abundant damage (Fig. 1C, D) in both weed species produced possibly by *Lysathiaflavipes* (Boheman, 1859) (Cordo and DeLoach 1982; Hernández and Cabrera Walsh 2014; Reddy et al. 2021a). On the other hand, an undescribed *Lysathia* was collected in 1991 from near the town of Penedo (Rio do Janeiro state, Brazil), and imported into quarantine in South Africa where it was studied and released as a biocontrol agent for *M.aquaticum* (Cilliers 1999b). It is important to note that while the collection site coordinates of this *Lysathia* sp. was originally reported as Penedo (10.1700S, 36.3600W) in Cilliers (1999b), these correspond to a different location, also called Penedo, in the Alagoas State. Based on confirmation from the original collection research team, the correct coordinates for Penedo, Rio do Janeiro, are 22.4383S, 44.5270W.

*Lysathiaflavipes*, initially studied by Cordo and DeLoach (1982) as a potential control agent for Ludwigiag.subsp.hexapetala, Ludwigiapeploidessubsp.montevidensis (Spreng.), and *M.aquaticum*, showed a limited host range to species of *Ludwigia* and *Myriophyllum.* Later, in the USA, Reddy et al. (2021a) investigated its biological parameters with a population collected in Uruguay, and concluded that this flea beetle was not a suitable candidate due to its lack of specificity. However, recent field observations showed morphological variability (e.g., variations in body and leg colouration, body size), its highly damaging capacity to the target species, and phylogenetic distance between these two main hosts suggest that *Lysathiaflavipes* could represent more than one species.

The unidentified species of *Lysathia*, published in earlier works as *Lysathia* sp., from now on the biocontrol (BC) agent *Lysathia* sp., has proven to be a highly effective biological control agent against *M.aquaticum* in South Africa for the past 30 years (Coetzee et al. 2011; Martin et al. 2018). Following its collection in 1991 (S. Neser, Plant Protection Research Institute, Pretoria, 1992 pers. comm.) David. G. Furth (Smithsonian Institute in Washington) analysed a sample of the beetle and established that it differed from the known species *L.flavipes* and *L.ludoviciana* (Fall, 1910), also found on the same host plant and suggested that the beetle likely represented a new species (Cilliers 1999b). Subsequent testing conducted under quarantine conditions at the ARC Plant Protection Research Institute revealed that *Lysathia* sp. fed exclusively on the emergent leaves of *M.aquaticum* and it was released in 1994. No other taxonomists were working on this genus and the beetle remained undescribed. Recent findings in Argentina (Misiones Province) of specimens collected on *M.aquaticum* that resemble the BC agent *Lysathia* sp., provided a new opportunity to describe this species.

Apart from *L.flavipes* and the BC agent *Lysathia* sp., *L.ludoviciana*, has been reported to feed on both Ludwigiag.subsp.hexapetala and *M.aquaticum* (Habeck and Wilkerson 1980). This species is considered native in North America, distributed from southeastern New York to Florida and westward to Texas and parts of Mexico (Chester and Holt 1990). It has been studied for its potential as a biological control agent in the USA; however, its efficacy may vary depending on the target plant species and environmental conditions and further research is needed (Campbell and Clark 1983; McGregor et al. 1996). Given the similarity in host plants, the lack of information available for the genus, and that *L.ludoviciana* is the only *Lysathia* species that has genetic information available in databases; it is an essential species that can contribute to both morphological and genetic comparisons in the search for unravelling the identities of *L.flavipes* and the BC agent *Lysathia* sp.

The genus *Lysathia* is taxonomically complex. It was proposed by Bechyné (1959) with seven species transferred from the genus *Altica* Geoffroy, 1762, with *L.flavipes* as the type species. The morphological characteristics upon which Bechyné based this separation were considered subtle by Scherer (1962), who subsequently combined *Lysathia* in his *Altica* key. However, as mentioned by Reid and Beatson (2015), Scherer’s action is not considered a nomenclatorial act and *Lysathia* remains a separate genus from *Altica*. As Bechyné (1959) described, *Lysathia* species are smaller than the ones catalogued within *Altica*, and characterised by the particularly incrassate femora, the feeble to sometimes indistinct basal transverse groove on the pronotum, and the male with a very short central lobe on the last abdominal sternite. Many species of *Lysathia* display a metallic body with fulvous legs, while in typical *Altica* the legs are usually metallic in colour. The aedeagus is of the same general type as in *Altica*, including the sculpture on its ventral side; however, rather large differences are observed in certain species, such as a strongly developed central ridge (Medvedev 2001). The genus includes species distributed in the Neotropic (South and Central America) and the south of the Nearctic region. According our bibliographical survey, there are 22 *Lysathia* species. Organised alphabetically, they are:

*L.aenea* (Oliver, 1808),
*L.arapata* Bechyné, 1959,
*L.atrocyanea* (Phil. & Phil., 1864),
*L.bohumilae* (Bechyné, 1954),
*L.chaparensis* Bechyné, 1959,
*L.comasagua* Bechyné & Bechyné, 1960,
*L.flavipes*,
*L.hygrobia* (Bechyné, 1955),
*L.integricollis* (Harold, 1876),
*L.jacobyi* (Csiki, 1939),
*L.louella* (Bechyné, 1955),
*L.ludoviciana*,
*L.muriensis* (Bechyné, 1954),
*L.occidentalis* (Suffrian, 1868),
*L.patagonica* Bechyné, 1957,
*L.philippi* (Csiki, 1939),
*L.rockefelleri* (Pallister, 1953),
*L.simplex* (Jacoby, 1891),
*L.siolii* Medvedev, 2001,
*L.viedma* Bechyné, 1957,
*L.virescens* (Blanchard, 1851),
*L.volcanica* Bechyné & Bechyné, 1960

(Bechyné 1957a, 1959; Bechyné and Springlová-Bechyné 1960, 1977; Furth and Savini 1996; Jerez 1988; Medvedev 2001; Scherer 1960; Virkki 1979). The species specifically distributed in Brazil and Argentina are *L.aenea*, *L.muriensis*, *L.siolii*, *L.bohumilae*, *L.flavipes*, *L.louella*, *L.viedma*, and *L.patagonica*.

To this date, there is no general systematic revisions focused on this genus and hence there is no current comprehensive key to differentiate these species. However, Bechyné (1954, 1955, 1959) contributed to the organisation of some of these species, particularly those distributed in southern South America, by referring to them in his publications as the *flavipes* group. Bechyné (1954) described two new *Altica* species, *A.bohumilae* and *A.muriensis*, grouping them with *A.flavipes*. Subsequently, in 1955, while describing additional *Altica* species from South America, he explicitly referenced *A.hygrobia* and *A.louella* as part of the “*A.flavipes* group” (Bechyné 1955). Since then, the term *flavipes* group has been used in publications, such as Bechyné (1959) including *L.arapata* and Medvedev (2001) with *L.siolii*. Other *Lysathia* species, such as *L.philippi*, *L.viedma*, and *L.patagonica*, are also described for southern South America but Bechyné does not include them in *flavipes* group since, apart from having a Patagonic distribution, they differ in morphological traits, such as having an opaque colouration and a different type of elytral punctation (Bechyné 1957a).

Precise taxonomic identification of biological control agents ensures that the selected species is effective against the target weed and does not pose risks to native or beneficial species. Misidentification of either the weed or the biological control agent can lead to ineffective control measures and unintended consequences such as damage to non-target species or ecosystems. The objective of this paper was to elucidate the inter- and intra-specific relationships of *L.flavipes* and the BC agent *Lysathia* sp. by performing phylogenetic analyses, subsequently, analysing these species through an integrative taxonomic approach, and establishing a one-to-one correlation between the morphological and genetic descriptions. In this study, the brief original description of *L.flavipes* is expanded, and the illustrations, distribution, and host plants updated. We also describe the BC agent *Lysathia* sp. against *M.aquaticum* as *Lysathiacilliersae* sp. nov.

## ﻿Materials and methods

### ﻿Study area and sample acquisition

Specimens (males and females) of *Lysathiaflavipes* were collected during several field trips across eastern and central Argentina where *Ludwigia* spp. and *Myriophyllumaquaticum* are most commonly distributed. Collections occurred between 2018 and 2023 in the Argentine provinces of Neuquén, Río Negro, Buenos Aires, Entre Ríos, Corrientes, and Misiones. Plants were inspected for characteristic damage caused by larvae or adults (Fig. 1C, D). Specimens were primarily collected as adults, except in locations where both plant species coexisted; in these cases, larvae were collected to ensure they were feeding on the host plant, then reared in laboratory until adults emerged. Also, preserved specimens of *L.flavipes* from Uruguay, evaluated in Reddy et al. (2021a), were included in the study. Since the BC agent *Lysathia* sp., originally sourced from Brazil, is used as a biological control agent against *M.aquaticum* in South Africa, specimens were obtained from a colony released in the 1990s from the Waainek mass rearing facility (Centre for Biological Control (CBC), Makhanda, South Africa). In addition, specimens suspected to be the same BC agent *Lysathia* species were collected in Misiones and Entre Ríos provinces (Argentina) while feeding on *M.aquaticum*. Lastly, preserved specimens of *L.ludoviciana* collected on *Ludwigia* sp. in Louisiana (USA) were obtained from a donation to the Coleoptera collection at the La Plata Museum.

All collected samples were preserved with 99% ethanol and stored at −20 °C. For each individual, a unique identification code, geographic coordinates (latitude and longitude), external physical characteristics (body and leg colour), sex, host plant, and collection date were recorded (Suppl. material 1: table S1). Specimens used for genetic and morphological analysis were dry-mounted and photographed using a Canon EOS 90D DSLR camera attached to a Leica S9 D stereomicroscope. Images were processed using focus stacking in Adobe Photoshop 2020.

### ﻿Genetic study

#### ﻿Genetic data

Individuals were selected for genomic DNA extraction using the Qiagen DNAeasy Blood & Tissue kit. To preserve reference specimens of each sample and allow for later correlation with their morphological descriptions, non-destructive extractions were performed using whole bodies, with a small puncture made in the left pleura using a fine needle. Specimens were subsequently recovered from the extraction process. Additionally, to increase DNA yield, a single left hind leg from each specimen was extracted and crushed. Only for two specimens from Uruguay, complete destruction of the specimens was necessary to enhance DNA extraction yield due to inadequate preservation. Two unlinked loci were sequenced (mitochondrial and ribosomal), to improve phylogenetic analyses. We were not successful in obtaining nuclear sequences. The Cytochrome c Oxidase Subunit I (COI) (~ 764 bp) and a ribosomal fragment corresponding to the 16S rRNA fragment (~ 524 bp), were amplified through polymerase chain reactions (PCR) in both directions. All samples were sequenced with primers C1J2195 (5’-TTGATT(CT)TTTGGTCA(CT)CC (AT)GAAGT-3’) and TL2N3014 (5’-TC(CT)A(AT)TGCA(CT) TAATCTGCCATATT-3’) (Simon et al. 1994). These primers have been previously used to amplify COI fragments of other flea beetles (Jenkins et al. 2009). Samples were also sequenced with 16sar-L (5’-CGCCTGTTTATC AAAAACAT-’3) and 16sbr-H (5’-CCGGTCTGAACTCAGATCAC-3’) primers after Salvi et al. (2019) targeting the fragment encompassing the domains IV and V of the16S rDNA. Details on PCR protocol are provided in the Suppl. material 1: PCR protocols. Sanger sequencing service from Macrogen was used.

In addition to the new sequences obtained, sequences previously published and available in the GenBank nucleotide database (https://www.ncbi.nlm.nih.gov/genbank/) were included. Specifically, the sequences downloaded were for *L.ludoviciana* from the mitochondrial COI gene (*n* = 10) and the ribosomal 16S (*n* = 2). Additionally, sequences from closely related taxa to *Lysathia* were retrieved to serve as outgroups: COI (*n* = 20) and 16S (*n* = 10); *Altica*, *Diorhabda*, *Longitarsus*, *Monolepta*, *Podontia*, *Psylliodes*, *Systena* and a specimen of the Galerucinae subfamily (likely *Paleosepharia* sp.) (Suppl. material 1: table S2). This study provides the first genetic sequences for *L.flavipes*. No previously published sequences were available for the analysis.

Sequences were inspected, trimmed, and aligned using GENEIOUS PRO v. 4.8 (http://www.geneious.com/), with the MUSCLE alignment algorithm (Edgar 2004). Each sequence was verified to correspond to a coding region, without gaps that could alter the reading frame. All new sequences obtained in this study were deposited in GenBank.

#### ﻿Phylogenetic reconstruction

Initially, a provisional morphological identification was conducted to group the collected specimens into the species to which they were suspected to belong. To examine the inter- and intra-specific phylogenetic relationships among the *Lysathia* species under study and to evaluate the correspondence between the obtained sequences and the morphological groupings, phylogenetic trees were constructed using new sequences from *L.flavipes*, the BC agent *Lysathia* sp., and *L.ludoviciana*. Sequences published in GenBank for *L.ludoviciana* and species from genera closely related to *Lysathia* were incorporated as outgroup. Several species of the genus *Altica* were specifically used to assess the proximity between the two genera, as some taxonomist question the separation of *Lysathia* proposed by Bechyné (1959). Phylogenetic trees were estimated for both individual and combined genes. Including a more conserved gene along with a more variable one can enhance branch resolution during reconstruction. Phylogenies were inferred using maximum likelihood (ML) analysis in RAxML-NG (Kozlov et al. 2019). The ML analysis was performed with bootstrap support values (BS) on 1,000 replications, using a GTRGAMMAI substitution model and standard parameters suggested by the software. Runs were conducted on the CERES HPC clusters server of the SCINet High-Performance Computer Systems of the ARS-USDA. The resulting trees were visualised using Figtree v. 1.4.4 software (http://tree.bio.ed.ac.uk/software/ﬁgtree/). Nodes with BS values exceeding 70% were considered sufficiently resolved (Hillis and Bull 1993).

#### ﻿Genetic diversity

To assess genetic variability within the species under study, *L.flavipes*, the BC agent *Lysathia* sp., and *L.ludoviciana*, a genetic diversity analysis was conducted using only mitochondrial data. Relationships between haplotypes were established by constructing a maximum parsimony network using the TCS inference algorithm implemented in PopArt v. 1.7. (Leigh and Bryant 2015), where haplotypes are connected by branches whose lengths are proportional to the number of mutations that differentiate them.

### ﻿Morphological study

We reviewed original descriptions and relevant literature for all species of the genus *Lysathia* (Olivier 1808; Blanchard 1851; Boheman 1859; Philippi and Philippi 1864; Harold 1876; Fall 1910; Heikertinger and Csiki 1939; Pallister 1953; Bechyné 1954, 1955, 1957a, 1959; Bechyné and Springlová-Bechyné 1960; Medvedev 2001; Peck et al. 2014). We focused on comparing the species of interest for this research with those inside the *flavipes* group or overlapping geographic distributions, as these were the most relevant candidates for potential misidentification. We also examined all available images of type species and specimens deposited at MLP.

In the morphological descriptions, traits shared between male and female are not repeated after their initial mention. Terminology for the cephalic capsule follows Konstantinov (1998b) and Reid and Beatson (2015); venation of the hind wing is based on Kukalová-Peck and Lawrence (1993), the metanotum and metaendosternite follow Konstantinov (1998b) and Lingafelter and Konstantinov (2000). Nomenclature of female and male genitalia is based on Konstantinov (1987, 1998a), Lingafelter and Konstantinov (2000), and Reid and Beatson (2015).

Measurements were taken with an ocular micrometre on an Olympus dissecting microscope and are expressed in millimetres (mm), with mean (X) and standard deviation (SD). Body measurements include: total length (from the base of the antennae to the tip of the elytron), elytral width at the height of the humeri, and maximum elytral width in the basal third. The drawings were made with a camera lucida on a Leitz compound microscope and a Wild dissecting microscope.

Specimens were compared with photographs of the type series of *L.flavipes* provided by the Swedish Museum of Natural History (Naturhistoriska riksmuseet, Stockholm-**NHRS**) under the Creative Commons Attribution 4.0 International Public License. Additionally, images of type specimens of *L.bohumilae* and *L.muriensis* Bechyné were provided by Frey Beetle Collection (Frey Naturhistorisches Museum Basel, Biowissenschaften). Finally, a bibliographical revision was conducted to locate any evidence of designation of the type series for *L.flavipes* with the aim to verify whether a lectotype had previously been designated or if the status of the series remained as originally described by Boheman (1859). This revision included searches in Zoological Records, Scopus, and relevant databases.

The studied specimens were deposited in the following institutions: Museo de La Plata (**MLP**), Buenos Aires, Argentina; Sala de Colecciones “Moisés Bertoni” (**SLP-art**), Misiones, Argentina; South African National Collection of Insects (**SANC**), Pretoria, South Africa; and the private collection of the Foundation for the Study of Invasive Species (**FuEDEI**).

## ﻿Results

### ﻿Study area and sample acquisition

**Figure 2.**
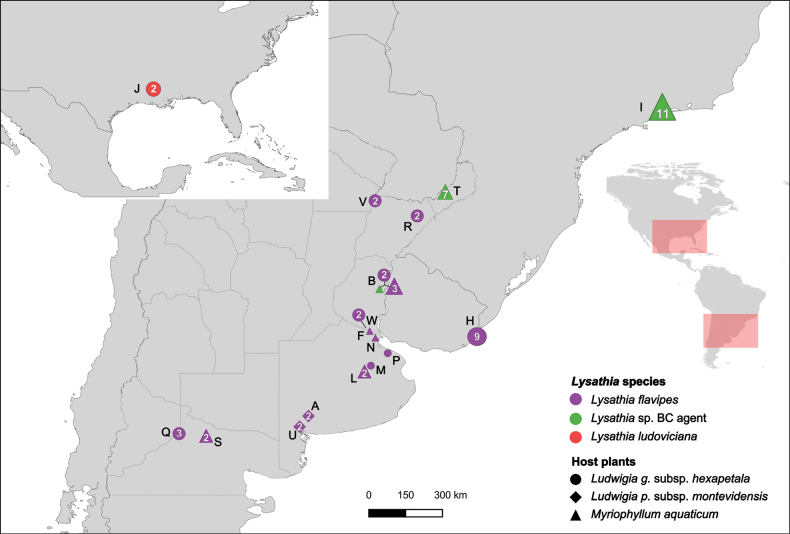
Collection sites for *Lysathia* specimens used in genetic and morphological studies. Colours on the map represent different *Lysathia* species, while shapes denote the host plants from which specimens were collected. Numbers within the shapes indicate the number of specimens, and letters correspond to the different localities. Additional information on localities are provided in Suppl. material 1: table S2.1.

The samplings conducted followed the distribution of the known host plants: Ludwigiagrandiflorasubsp.hexapetala, L.peploidessubsp.montevidensis, and *Myriophyllumaquaticum* in Argentina (Fig. 2).

A total of 35 specimens, initially assigned to *L.flavipes*, were collected from the central-east region of Argentina (from the northeast of Corrientes to the northernmost part of Patagonia). Additionally, nine specimens collected in Uruguay and studied in Reddy et. al (2021a), were donated from collaborators. For the BC agent *Lysathia* sp., 11 specimens were obtained from the mass rearing centre in South Africa (plotted at their origin site in Brazil, in Fig. 2) and eight specimens from Argentina. Lastly, two samples of *L.ludoviciana* were obtained from MLP. As a result, a total of 65 specimens were considered in our study.

### ﻿Genetic study

#### ﻿Phylogenetic analyses

Of the 65 specimens from which DNA was extracted, 47 yielded successful sequences of the mitochondrial COI (presumed *L.flavipes* [*n* = 23], *L.ludoviciana* [*n* = 2], and presumed BC agent *Lysathia* sp. [*n* = 17]). Of the ribosomal 16S gene, 23 sequences were obtained (*L.flavipes* [*n* = 10], *L.ludoviciana* [*n* = 2], and BC agent *Lysathia* sp. [*n* = 11]) (Table 1).

**Table 1.** *Lysathia* specimens used in genetic analyses. Sample: respective sample codes; Species: initially assigned species, *Lysathiaflavipes*, *L.ludoviciana* and the biocontrol (BC) agent *Lysathia* sp.; Sex: ♂= male ♀= female; Host plant: *Ludwigiap.* subsp. montevidensis, *L.g*. subsp. hexapetala and *M.aquaticum*; Sites of collection with their respective Code (letters), locality (Loc.), province (Prov.), and Country (ARG = Argentina, UY = Uruguay, SA = South Africa, and USA = United States of America). Accession number with which the sequences were entered into GenBank for Cytochrome Oxidase I (COI) and 16S ribosomal. (*) indicates that the specimen was later found not to be the initially assigned species.

Sample	Species	Sex	Host Plant	Site		GenBank	
				Code	Loc., Prov., Country	COI	16S
LM_A_02	*L.flavipes*	♂	*L.p.montevidensis*	A	V. Ventana, BA, ARG	PQ558504	
LH_B_01	*L.flavipes*	♂	*L.g.hexapetala*	B	Calabacillas, ER, ARG	PQ558491	
LH_B_02	*L.flavipes*	♀	*L.g.hexapetala*	B	Calabacillas, ER, ARG	PQ558492	PQ621632
MA_B_01	*L.flavipes*	♂	*M.aquaticum*	B	Calabacillas, ER, ARG	PQ558507	
MA_B_02	*L.flavipes*	♀	*M.aquaticum*	B	Calabacillas, ER, ARG	PQ558508	PQ621637
MA_B_03	*L.flavipes*	♂	*M.aquaticum*	B	Calabacillas, ER, ARG	PQ558509	PQ621638
MA_F_01	L.flavipes*	♀	*M.aquaticum*	F	Otamendi, BA, ARG	PQ558510	PQ621639
LH_H_08	*L.flavipes*	♀	*L.g.hexapetala*	H	P. del Diablo, RO, UY	PQ558493	
LH_H_09	*L.flavipes*	♂	*L.g.hexapetala*	H	P. del Diablo, RO, UY	PQ558494	
MA_L_01	*L.flavipes*	♂	*M.aquaticum*	L	Las Flores, BA, ARG	PQ558511	PQ621640
MA_L_02	*L.flavipes*	♀	*M.aquaticum*	L	Las Flores, BA, ARG	PQ558512	
LH_M_01	L.flavipes*	♀	*L.g.hexapetala*	M	Gorchs, BA, ARG	PQ558495	PQ621633
LH_P_01	*L.flavipes*	♀	*L.g.hexapetala*	P	La Plata, BA, ARG	PQ558496	
LH_Q_01	*L.flavipes*	♀	*L.g.hexapetala*	Q	Plottier, NQ, ARG	PQ558497	
LH_Q_02	*L.flavipes*	♀	*L.g.hexapetala*	Q	Plottier, NQ, ARG	PQ558498	
LH_Q_03	*L.flavipes*	♂	*L.g.hexapetala*	Q	Plottier, NQ, ARG	PQ558499	
LH_R_01	*L.flavipes*	♂	*L.g.hexapetala*	R	Galarza, CR, ARG	PQ558500	PQ621634
MA_S_02	*L.flavipes*	♀	*M.aquaticum*	S	V. Regina, RN, ARG	PQ558513	
LM_U_01	*L.flavipes*	♀	*L.p.montevidensis*	U	B. Blanca, BA, ARG	PQ558505	
LM_U_02	*L.flavipes*	♂	*L.p.montevidensis*	U	B. Blanca, BA, ARG	PQ558506	
LH_V_01	*L.flavipes*	♂	*L.g.hexapetala*	V	L. Brava, NQ, ARG	PQ558501	PQ621635
LH_V_02	*L.flavipes*	♀	*L.g.hexapetala*	V	L. Brava, CR, ARG	PQ558502	
LH_W_02	*L.flavipes*	♂	*L.g.hexapetala*	W	B. Largo, ER, ARG	PQ558503	PQ621636
MA_I_01	*L.* sp. BC agent	♀	*M.aquaticum*	I	Makhanda, EC, SA	PQ558476	PQ621621
MA_I_02	*L.* sp. BC agent	♂	*M.aquaticum*	I	Makhanda, EC, SA	PQ558477	PQ621622
MA_I_03	*L.* sp. BC agent	♀	*M.aquaticum*	I	Makhanda, EC, SA	PQ558478	PQ621623
MA_I_04	*L.* sp. BC agent	♀	*M.aquaticum*	I	Makhanda, EC, SA	PQ558479	
MA_I_05	*L.* sp. BC agent	♂	*M.aquaticum*	I	Makhanda, EC, SA	PQ558480	PQ621624
MA_I_06	*L.* sp. BC agent	♂	*M.aquaticum*	I	Makhanda, EC, SA	PQ558481	
MA_I_07	*L.* sp. BC agent	♀	*M.aquaticum*	I	Makhanda, EC, SA	PQ558482	PQ621625
MA_I_08	*L.* sp. BC agent	♂	*M.aquaticum*	I	Makhanda, EC, SA	PQ558483	PQ621626
MA_I_09	*L.* sp. BC agent	♂	*M.aquaticum*	I	Makhanda, EC, SA	PQ558484	PQ621627
MA_I_10	*L.* sp. BC agent	♀	*M.aquaticum*	I	Makhanda, EC, SA	PQ558485	PQ621628
MA_I_11	*L.* sp. BC agent	♀	*M.aquaticum*	I	Makhanda, EC, SA	PQ558486	PQ621629
MA_T_01	*L.* sp. BC agent	♂	*M.aquaticum*	T	D. Savio, MS, ARG	PQ558487	PQ621630
MA_T_02	*L.* sp. BC agent	♂	*M.aquaticum*	T	D. Savio, MS, ARG	PQ558488	PQ621631
MA_T_03	*L.* sp. BC agent	♂	*M.aquaticum*	T	D. Savio, MS, ARG	PQ556198	
MA_T_04	*L.* sp. BC agent	♀	*M.aquaticum*	T	D. Savio, MS, ARG	PQ556199	
MA_T_05	*L.* sp. BC agent	♂	*M.aquaticum*	T	D. Savio, MS, ARG	PQ558489	
MA_T_07	*L.* sp. BC agent	♂	*M.aquaticum*	T	D. Savio, MS, ARG	PQ558490	
MA_J_01	*L.ludoviciana*	♀	*Ludwigia sp.*	J	Kaplan, LU, EEUU	PQ558514	PQ621641
MA_J_02	*L.ludoviciana*	♂	*Ludwigia sp.*	J	Kaplan, LU, EEUU	PQ558515	PQ621642

Although DNA amplification of the nuclear Wingless gene was achieved, the obtained sequences exhibited very low signal in the chromatogram, making their accurate interpretation difficult. Consequently, these sequences were not considered reliable, and the use of this locus was discarded. Therefore, including the sequences obtained from GenBank, the final alignment consisted of 52 total sequences for COI and 23 for 16S.

**Figure 3.**
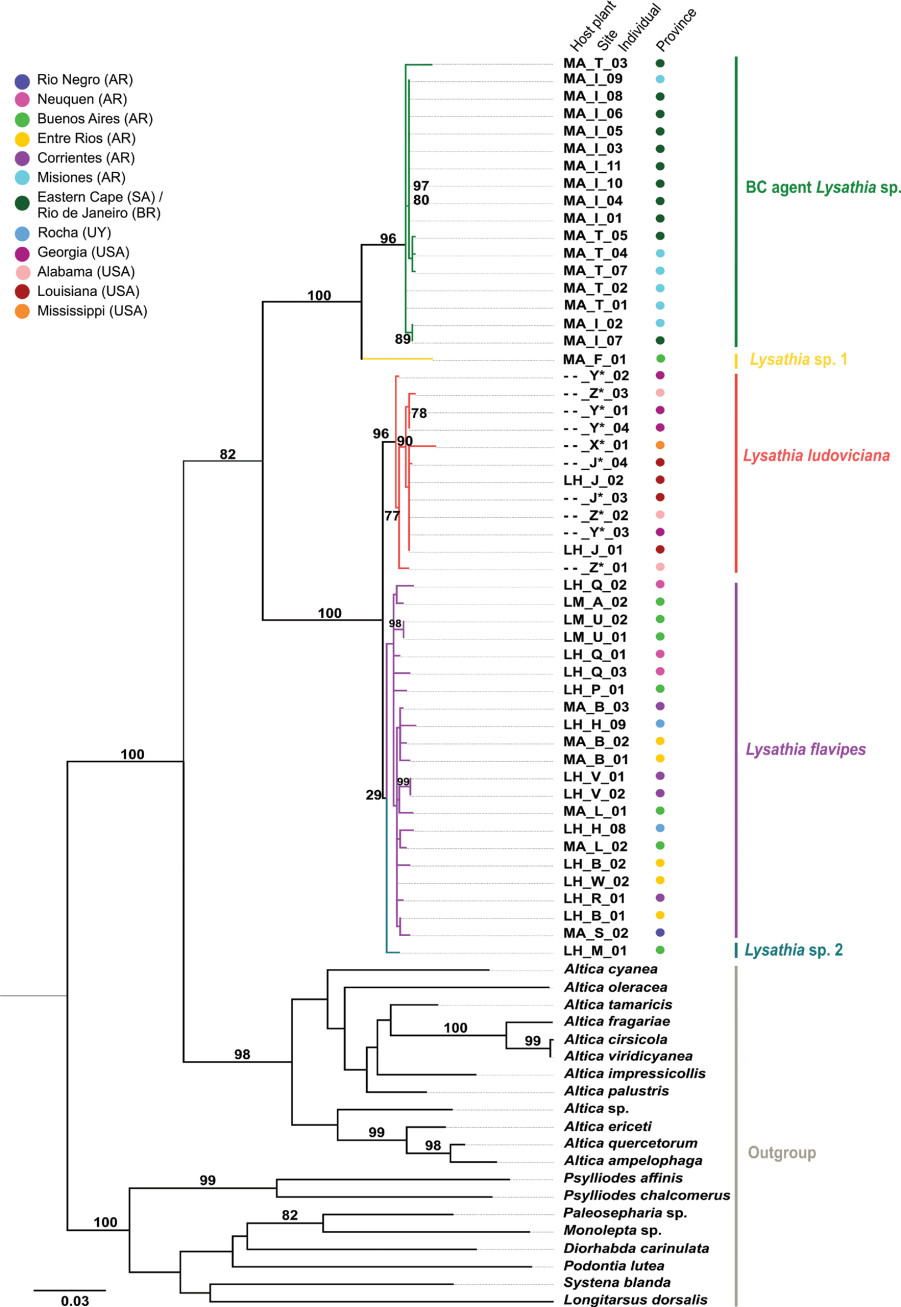
Best tree generated in RAxML for the *Lysathia* species studied, based on the mitochondrial Cytochrome Oxidase I (COI) gene. Names at the end of each terminal indicate the collection code (host plant_collection site code_number of individual). Abbreviation of host plants: *Myriophyllumaquaticum* (MA), Ludwigiag.subsp.hexapetala (LH), Ludwigiap.subsp.montevidensis (LM), and unknown plant (--). Collection site code: see Table 1. The coloured circles refer to the provinces/states where the specimens were collected. Specimens marked with * correspond to sequences obtained from GenBank.

**Figure 4.**
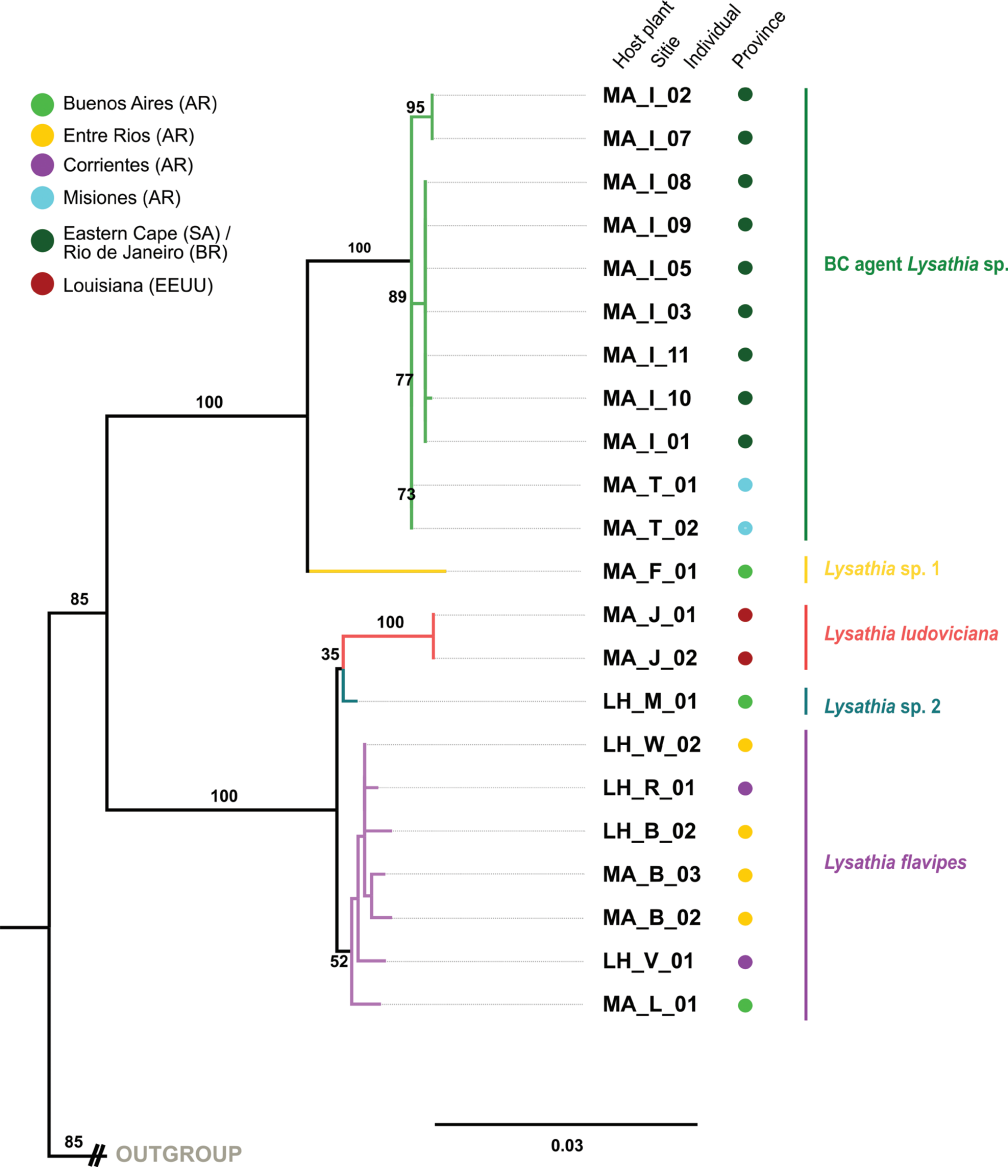
Best tree generated in RAxML for the *Lysathia* species studied, based on the combined mitochondrial (COI) and ribosomal (16S) genes. Names at the end of each terminal indicate the collection code (host plant_collection site code_number of individual). Abbreviation of host plants: *Myriophyllumaquaticum* (MA), Ludwigiag.subsp.hexapetala (LH), Ludwigiap.subsp.montevidensis (LM). Collection site code: see Table 1. The coloured circles refer to the provinces/states where the specimens were collected. The outgroup has been removed for clarity. The complete tree is presented in Suppl. material 1: figure S4.2.

The tree estimated using RAxML based on the mitochondrial COI gene fragment (Fig. 3) showed a higher level of discrimination compared to the ribosomal 16S tree (Suppl. material 1: fig. S1). The samples of the genus *Altica* used as an outgroup were deeply rooted in the tree, with high bootstrap support (BS) value of 98% at the nodes separating them from the *Lysathia* species (Fig. 3). This high support was also observed for the other genera evaluated as outgroups (BS = 100%). The phylogenetic tree revealed that the *Lysathia* species studied form a well-defined group which does not contradict the hypothesis of monophyly for the genus (BS = 82%). The collected specimens were organised into two main clades, one comprising the BC agent *Lysathia* sp. and *Lysathia* sp. 1, and the other consisting of *L.ludoviciana*, *L.flavipes*, and *Lysathia* sp. 2. Originally identified as *L.flavipes*, *Lysathia* sp. 1 and sp. 2 were reclassified due to their distinct grouping from *L.flavipes*. Both the mitochondrial gene tree and the combined tree with the ribosomal gene, showed well-resolved branch values (BS = 100%) (Figs 3, 4).

The specimens collected as *L.flavipes* (purple subclade), despite exhibiting morphological variations, different host plants, and diverse geographical distribution, were mostly grouped into a single subclade with the exception of the two specimens, *Lysathia* sp. 1 and *Lysathia* sp. 2. This subclade also included the specimens from Uruguay, evaluated in the USA as potential biological control agents. The phylogenetic support for *L.flavipes* was weak (BS = 29%) (Fig. 3) although it was consistent for both genes and improved in the combined tree (BS = 52%) (Fig. 4). *Lysathia* sp. 1, is a single specimen from Otamendi, Buenos Aires. In both trees, it was positioned as a sister species to the BC agent *Lysathia* sp. with good support (Figs 3, 4). Conversely, *Lysathia* sp. 2, from Gorchs (Buenos Aires) showed uncertain placement. In the mitochondrial gene tree, *Lysathia* sp. 2 was positioned as a sister species to *L.flavipes* (Fig. 3), while in the combined tree, it positioned as a sister species to *L.ludoviciana* (Fig. 4).

The specimens of *L.ludoviciana*, both samples obtained from GenBank and the newly sequenced ones, grouped into a well-defined subclade with a high BS support value (96%; Fig. 3, red colour). This indicates strong phylogenetic support for the morphological correspondence of this group. In the combined tree, although based on fewer specimens, good branch support was also obtained (Fig. 4). In both cases, *L.ludoviciana* was the species phylogenetically closest to *L.flavipes* compared to the BC agent *Lysathia* sp.

The specimens identified as the BC agent *Lysathia* sp., including those from the mass rearing centre in South Africa and originally from Penedo (Rio de Janeiro, Brazil), as well as those collected in Misiones (Argentina), grouped into a single subclade with high BS support value (COI: BS = 96%; COI+16S: BS = 99%) (Figs 3, 4, green clade). This strong phylogenetic support indicates that specimens from both locations (Rio de Janeiro and Misiones) belong to the same species, the BC agent *Lysathia* sp.

#### ﻿Genetic diversity

**Figure 5.**
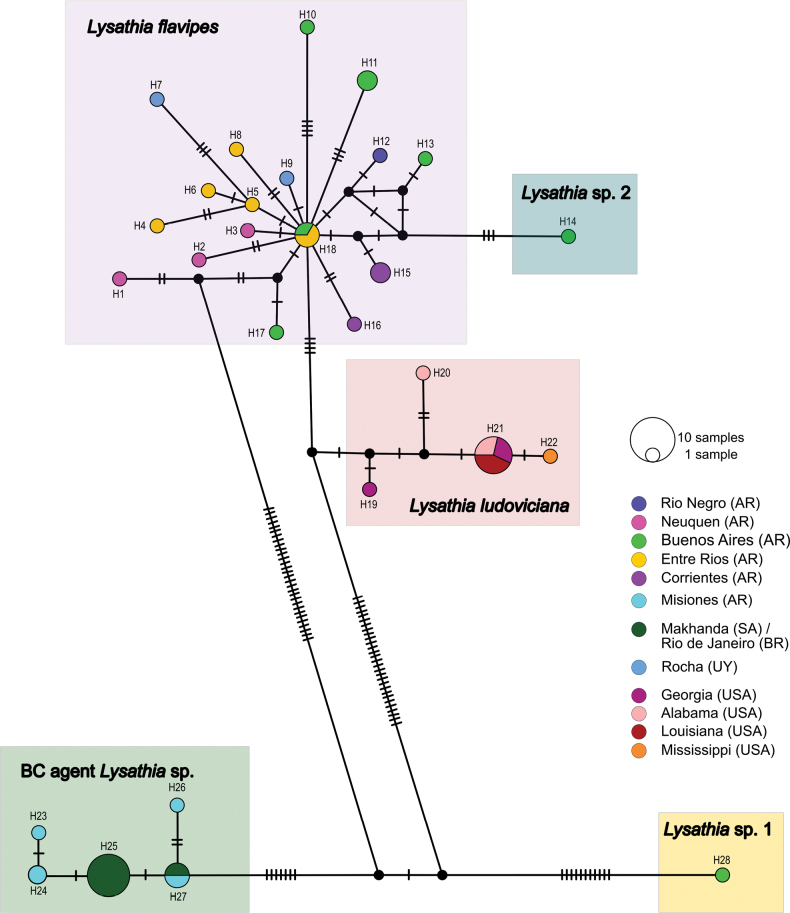
Mitochondrial haplotype network (COI) for *Lysathia* species estimated using the TCS algorithm in PoPArt. Each circle represents a haplotype, with its size proportional to its frequency. Colours indicate the province or state where the specimens were collected.

Of the 53 sequences analysed, 28 haplotypes were identified, grouped into five clusters, three of which correspond to the *Lysathia* species studied (Fig. 5). The cluster associated to the specimens collected as *L.flavipes* consisted of 22 sequences and 18 haplotypes, demonstrating considerable haplotype diversity.

The H18 haplotype, consisting of specimens from Buenos Aires and Entre Ríos provinces (Fig. 5), appears to be the most ancestral within the cluster due to its multiple connections with other haplotypes and its central position. Similar to observations in the phylogenetic trees (Figs 3, 4), the H14 haplotype, represented by a specimen collected in Gorchs (Buenos Aires Province), shows distinctiveness, suggesting it may represent a distinct or new species (*Lysathia* sp. 2).

**Figure 6.**
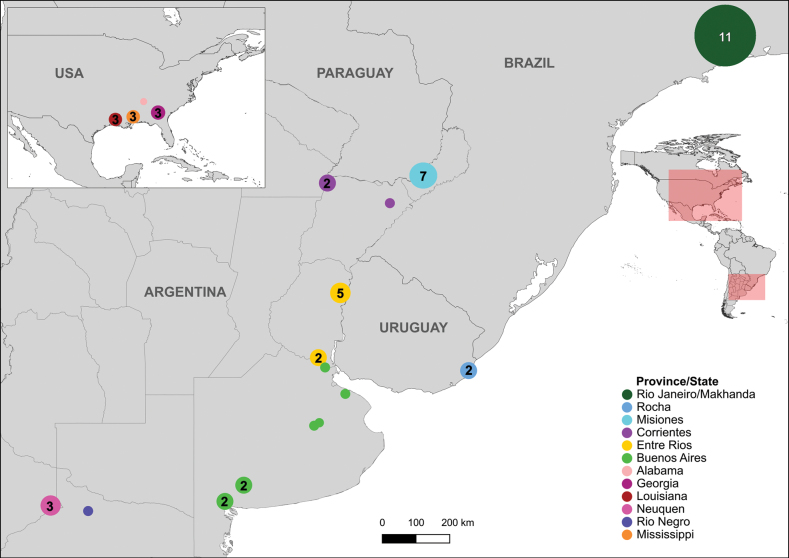
Map showing collection sites where DNA samples of *Lysathia* spp. were obtained. Circles represent collection sites, with their size proportional to the number of sequences collected (numbers inside the circles). Colours represent provinces or states where samples were collected, matching those shown in the haplotype network. Additional details are provided in Table 1.

The cluster corresponding to specimens collected as the BC agent *Lysathia* sp. consisted of 17 sequences and five haplotypes. This cluster includes specimens from Misiones and South Africa (derived from insects collected in Rio de Janeiro, Brazil), which supports the observed morphological similarity in the field among these specimens. Notably, haplotype H27 was found in specimens from both locations (Fig. 6), suggesting they belong to the same species.

The cluster comprising specimens collected as *L.ludoviciana* consisted of ten sequences and four haplotypes. Among these, haplotype H21 was the most frequent, including specimens distributed in Louisiana, Alabama, and Georgia. The haplotype network indicates a close relationship between *L.flavipes* and *L.ludoviciana*.

Finally, a unique haplotype (H28) from Buenos Aires Province (Otamendi locality) was observed. This haplotype, originally identified as a specimen of *L.flavipes*, did not group with other *L.flavipes* haplotypes and was thus reclassified as *Lysathia* sp. 1. Haplotype H28 was found to be highly differentiated from the clusters of *L.flavipes* and *L.ludoviciana* and, to a lesser extent, although still highly differentiated, from the BC agent *Lysathia* sp. This observation, supported by the phylogenetic study, suggests that *Lysathia* sp. 1 likely represents a distinct species from the three evaluated in this analysis.

#### ﻿Morphological studies

Previous knowledge of the morphology of some of these species, combined with the results from phylogenetic reconstruction and genetic diversity analysis of collected specimens, provided robust support for the morphological characterisation of *L.flavipes* and the BC agent *Lysathia* sp. Since all specimens used for genetic studies were preserved, the 47 individuals were morphologically compared. For *L.flavipes*, this comparison allowed for the incorporation of new information to expand upon Boheman’s (1859) brief description. For the BC agent *Lysathia* sp., the morphological analysis enabled us to provide a detailed description of a new species, *Lysathiacilliersae* sp. nov.

### ﻿Taxonomic account

#### ﻿ Lysathia flavipes

Taxon classificationAnimaliaColeopteraChrysomelidae

(Boheman, 1859)

CC0ABE59-CF6B-57D6-97EF-B53812CADAC6

Figs 7 , 8 , 9 , 10

Graptodera flavipes Boheman, 1859: 188.
Altica flavipes (Boheman): Weise 1888: 825.
Lysathia flavipes (Boheman), Bechyné 1959: 303.

##### Lectotype designation.

Boheman (1859) did not specify a holotype specimen or number of specimens included in the original type series. To stabilise the nomenclature and provide a clear reference point, we designate here as the lectotype the female specimen labelled NHRS-JLKB 000073986 from Montevideo, Uruguay, housed at the Swedish Museum of Natural History. The remaining specimens from the original series are hereby designated as paralectotypes: NHRS-JLKB 000073987 (from Montevideo, Uruguay), NHRS-JLKB 000073988 (from Buenos Aires, Argentina), and NHRS-JLKB 000073989 (also from Buenos Aires, Argentina).

##### Material examined.

**Figure 7.**
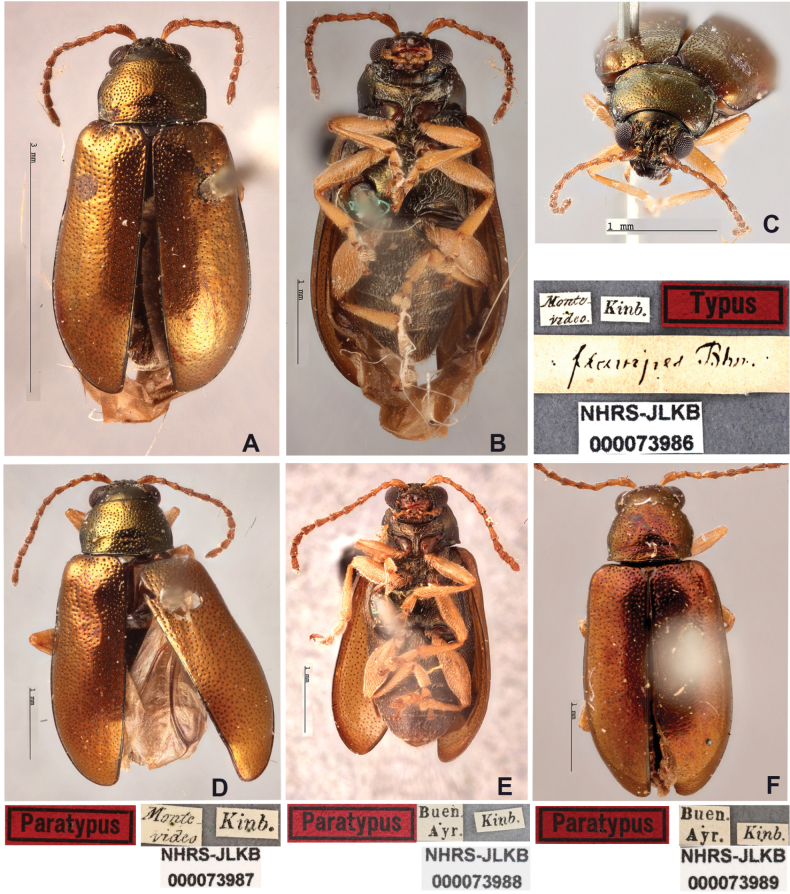
*Lysathiaflavipes* (Boheman) **A–C** lectotype, female **A** dorsal **B** ventral **C** head **D** paralectotype, Montevideo **E** paralectotype Buenos Aires, male, ventral **F** paralectotype Buenos Aires, male, dorsal. Photographed by Anna Jerve (© 2022 Naturhistoriska Riksmuseet). Original photographs provided by the Swedish Museum of Natural History under Creative Commons Attribution 4.0 International Public License, CC-BY 4.0 (https://creativecommons.org/licenses/by/4.0/legalcode).

***Lectotype***: Photographs of the lectotype with labels. Uruguay • 1♀, pinned; Montevideo; 1859; “Kinb.”, “flavipes Bhn.; NHRS-JLKB000073986. ***Paralectotypes***: Photographs of Paralectotypes with lables. Argentina • 1♂ pinned; “Buen. Ayr.” [Buenos Aires]; “Kinb.”; NHRS-JLKB000073988 • 1♂, pinned; “Buen. Ayr.” [Buenos Aires]; “Kinb.”; NHRS-JLKB000073989. Uruguay • 1♀, pinned; Montevideo; “Kinb.”; NHRS-JLKB000073987 (Fig. 7).

##### Other material examined.

Specimens used in genetic and morphological studies. Argentina – **Buenos Aires prov.** • ♀; Bahía Blanca; -38.699335, -62.329274; 28 Feb. 2021; A. Faltlhauser leg.; on *Ludwigiap.* subsp. montevidensis, chemically treated for DNA extraction; GenBank: PQ558505; Collectors code (C.c.): LFLMU01 (47); MLP • ♂; same data as preceding; GenBank: PQ558506; C.c.: LFLMU02 (48); MLP • ♂; Villa Ventana; -38.069774, -61.925423; 28 Feb 2021; A. Faltlhauser leg.; on *Ludwigiap.* subsp. montevidensis, chemically treated for DNA extraction; GenBank: PQ558504; C.c.: LFLMA02 (38); MLP • ♀; same locality and date as preceding; A. Faltlhauser leg.; on *Ludwigiap.* subsp. montevidensis; C.c.: LFLMA01 (16); MLP • ♂; Las Flores; -35.955143, -59.011231; 6 May 2022; A. Faltlhauser leg.; on *Myriophyllumaquaticum*, chemically treated for DNA extraction; GenBank: PQ558511, PQ621640; C.c.: LFMAL01 (12); MLP • ♀; same data as preceding; GenBank: PQ558512; C.c.: LFMAL02 (35); MLP • ♀ La Plata; -35.002000, -58.0256321; 21 Dic 2021, A. Faltlhauser leg; on *Ludwigiag.* subsp. hexapetala, chemically treated for DNA extraction; GenBank: PQ558496; C.c.: LFLHP01 (8); MLP • ♀ Dique Luján; -34.36496, -58.67688; 23 Sep 2021; on *Myriophyllumaquaticum*, chemically treated for DNA extraction; C.c.: LFMAN01 (10); MLP • ♀; R. Otamendi; -34.098854, -58.796713; 26 Jan 2021; A. Faltlhauser leg.; on *Myriophyllumaquaticum*, chemically treated for DNA extraction; GenBank: PQ558510, PQ621639; C.c.: LFMAF01 (11); MLP. – **Corrientes prov.** • ♂; Galarza; -28.090452, -56.709842; 01 Dic 2021; A. Faltlhauser leg; on *Ludwigiag.* subsp. hexapetala, chemically treated for DNA extraction; GenBank: PQ558500, PQ621634; C.c.: LFLHR01 (13); MLP • ♀; same data as preceding; C.c.: LFLHR02 (39); MLP • ♂; Laguna Brava; -27.49034, -58.71573; 29 May 2022; A. Faltlhauser leg; on *Ludwigiag.* subsp. hexapetala, chemically treated for DNA extraction; GenBank: PQ558501, PQ621635; C.c.: LFLHV01 (14); MLP • ♀; same data as preceding; GenBank: PQ558502; C.c.: LFLHV02 (19); MLP. – **Entre Ríos prov.** • ♂; Calabacillas, Cañada Venancio; -31.54152, -58.18974; 11 Jan 2022; A. Faltlhauser leg; on *Ludwigiag.* subsp. hexapetala, chemically treated for DNA extraction; GenBank: PQ558491; C.c.: LFLHB01 (40); MLP • ♀; same data as preceding; GenBank: PQ558492, PQ621632; C.c.: LFLHB02 (41); MLP • ♂; same location and collector as preceding; on *Myriophyllumaquaticum*, chemically treated for DNA extraction; GenBank: PQ558507; C.c.: LFMAB01 (42); MLP • ♀; same data as preceding; GenBank: PQ558508, PQ621637; C.c.: LFMAB02 (43); MLP • ♂; same data as preceding; GenBank: PQ558509, PQ621638; C.c.: LFMAB03 (44); MLP • ♀; Brazo Largo; -33.865303, -58.88294; 5 Feb 2022; A. Faltlhauser leg; on *Ludwigiag.* subsp. hexapetala, chemically treated for DNA extraction; C.c.: LFLHW01 (15); MLP • ♂; same data as preceding; GenBank: PQ558503, PQ621636; C.c.: LFLHW02 (20); MLP. – **Neuquén prov**. • ♀; Plottier, Río Limay; -38.969486, -68.185343; 21 Feb 2021; A. Faltlhauser leg; on *Ludwigiag.* subsp. hexapetala, chemically treated for DNA extraction; GenBank: PQ558497; C.c.: LFLHQ01 (9); MLP • ♂; same data as preceding; GenBank: PQ558498; C.c.: LFLHQ02 (36); MLP • ♂; same data as preceding; GenBank: PQ558499; C.c.: LFLHQ03 (37); MLP. – **Río Negro prov.** • ♂; Villa Regina; -39.09821, -67.075377; 29 Mar 2022; A. Faltlhauser leg.; on *Myriophyllumaquaticum*, chemically treated for DNA extraction; C.c.: LFMAS01 (31); MLP • ♀; same data as preceding; chemically treated for DNA extraction; GenBank: PQ558513; C.c.: LFMAS02 (32), MLP. Uruguay – **Rocha prov.** • 5♀, 2♂; Punta del Diablo; -34.0130556, -53.598305; 10 Mar 2019; P. Pratt leg.; on *Ludwigiag.* subsp. hexapetala, chemically treated for DNA extraction; C.c.: LFLHH01-07 (17,18,21–23,33,34); MLP • ♀; same data as preceding; GenBank: PQ558493; C.c.: LFLHH08 (UY1) • ♂; same data as preceding; GenBank: PQ558494; C.c.: LFLHH09 (UY2).

##### Diagnosis.

The characters that identify this species are: predominant bronze colour with golden, greenish, or purple reflections and piceous abdomen. Antennae brown, antennomere 1–3 yellowish. Antennomere 2 oval. Antebasal sulcus of prothorax slightly impressed. Lamina of sternite 8 subtriangular, spatular or with irregularly expanded sides; vaginal palpi short, conical. Median lobe broadened anteriorly from medial length, margins of dorsal depression with small denticles.

##### Description.

**Figure 8.**
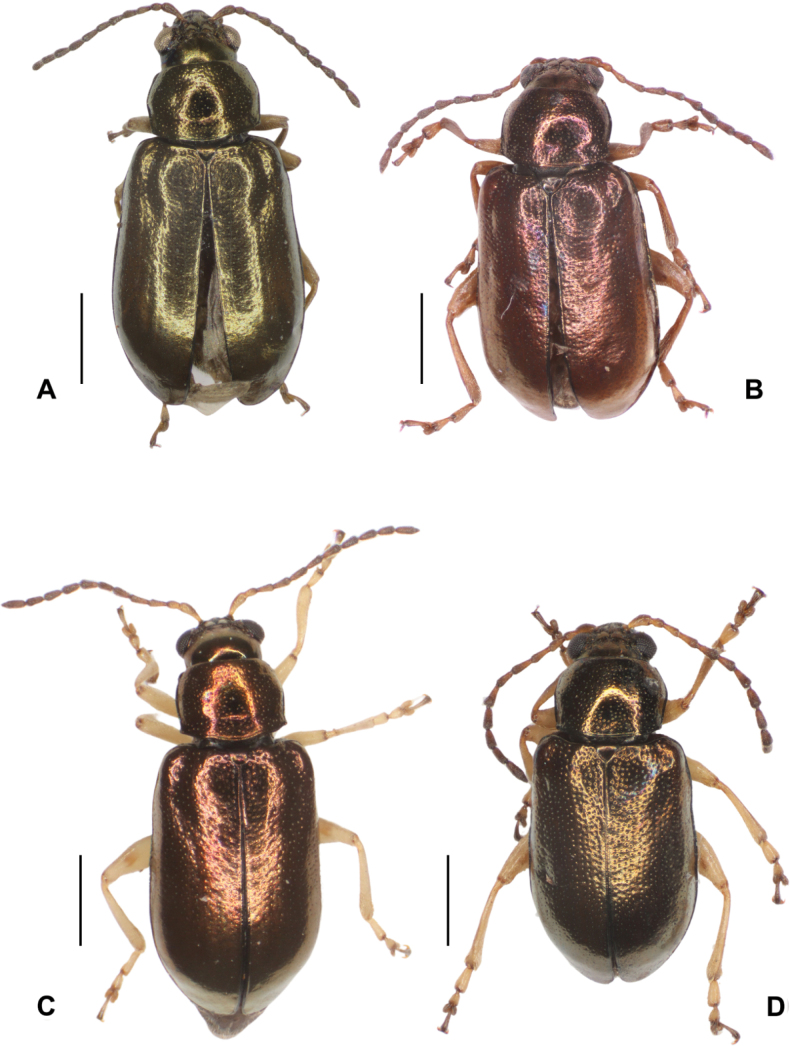
*Lysathiaflavipes*, examples of colour variability **A** bronze-piceous dorsum (code MA_N_01) with a golden-green metallic sheen. Female. Dique Luján, Argentina **B** purplish piceous dorsum. Legs pale brown (code LM_U_01). Female. Bahía Blanca, Arg **C** copper-piceous dorsum. Whitish yellow legs. Female. Dique Luján, Arg **D** bronze -piceous dorsum. Legs whitish yellow. Male. La Plata, Arg.

**Figure 9.**
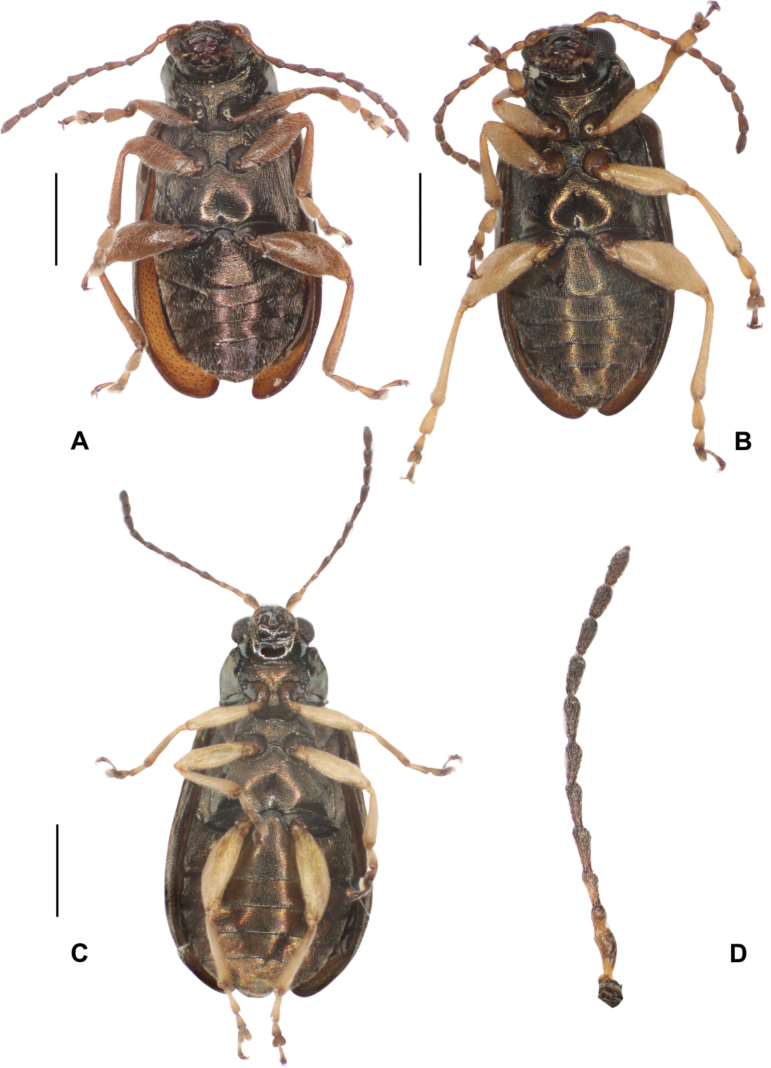
*Lysathiaflavipes*, ventral, piceous colour. Variability in leg colouration **A** pale brown legs (code LM_U_01). Female. Bahía Blanca, Arg **B** whitish yellow legs. Male. La Plata, Arg **C** whitish yellow legs. Female. Dique Luján, Arg **D** left antenna. Female, La Plata, Arg.

**Figure 10.**
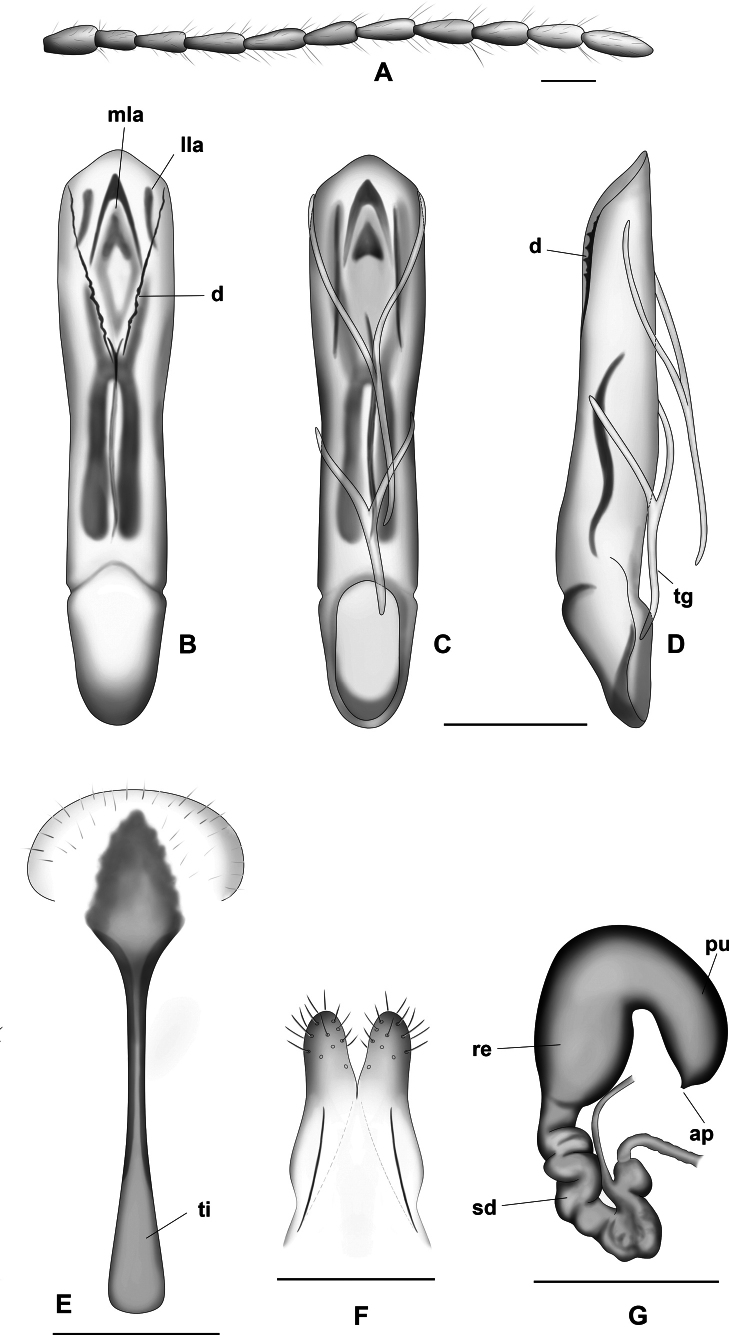
*Lysathiaflavipes***A** antenna **B–D** median lobe of aedeagus **B** dorsal view **C** ventral view **D** lateral view **E** sternite 8 **F** vaginal palpi **G** spermatheca. Abbreviations: **ap**: appendix, **d**: denticles, **lla**: lateral lamella, **mla**: median lamella, **pu**: pump, **re**: receptacle, **sp**: spermathecal duct, **tg**: tegmen, **ti**: tignum. Illustrations by Dr. Julia Rouaux. Scale bars: 0.1 mm.

**Female.** The description is based on photographs of the lectotype and paralectotypes, and on specimens collected in Argentina and Uruguay. Length: 4.3 (0.13); elytral base width: 1.7 (0.04); max. elytral width: 2.1 (0.13) (*n* = 7). ***Colour*.** Head bronze. Antennae brown, antennomeres 1–3 pale brown or yellowish. Mandibles, labium, and maxillae brown. Scutellum piceous. Thorax and dorsal elytra bronze with golden, greenish, or purple reflections. Abdomen, piceous. Legs whitish yellow, coxae chestnut, tarsomeres 1–3 yellowish, distal part dark brown. Ventral surface piceous (Figs 7–9). ***Head*** (Fig. 7C). Vertex glabrous, coarse setiferous punctures located above posterior margin of eyes, others located between inner margin of eyes and antennal calli. Post antennal calli barely raised, trapezoidal; antennal sockets closed to anterior margin of eyes, distance between them greater than transverse diameter of antennal sockets. Frontal ridge moderately convex; anterofrontal ridge lower than frontal ridge in lateral view, with small setae on each side, two or three short setae below antennal sockets and two long setae near base of clypeus. Antennae 11-segmented, inserted below midline of eyes, extended less than half of elytral length; antennomere 2 short, oval, longer than half the length of antennomere 3, antennomere 3 elongate, barely shorter than antennomere 4; antennomeres 4–7 cylindrical, similar in length, antennomeres 8–10 somewhat shorter, antennomere 11 acuminate at apex. Antennomeres 1–3 scarcely setose, from 4–11 densely setose over entire surface; all antennomeres with stiff, spreading setae at apex (Figs 9D, 10A). Eyes convex, inner margin slightly concave. ***Thorax*** (Figs 7, 8). Pronotum slightly convex, with irregular punctation, maximum width near mid-width. Antebasal sulcus slightly impressed, not reaching lateral margins; anterior lateral angles rounded, posterior angles acuminate, each with a long seta. Prosternum convex, finely pubescent, narrow prosternal process, rounded at apex, extending beyond the procoxal cavities; these are rounded and open posteriorly. Mesosternum with dense pubescence, short and wide mesosternal process; mesocoxal cavities inserted at the posterior margin. Metasternum usually longer than wide, central area finely striated, densely pubescent laterally, metacoxal cavities transverse, inserted at the posterior margin. Elytra convex, broader than pronotum; maximum width near the posterior third. Surface uniformly punctate in the basal half, with punctures similar but sparser than those on the pronotum, and more finely punctate towards the apex. Discal punctures aligned in two short rows near the elytral suture, humeral calli rounded, smooth. Epipleura subvertical, broad at the base, gradually narrowed towards the middle of the elytral length. Legs with the pro-mesofemora fusiform, moderately broadened; metafemora broader, dorsal margin slightly convex, anterior margin nearly straight. Apical margins of all tibiae with short spurs; trochanters, femora, and tibiae of all legs sparsely setose; ventral and lateral margins of metalegs densely setose. Tarsomere 1 of prolegs triangular, shorter than tarsomeres 2+3 combined; tarsomere 2 triangular; tarsomere 3 deeply bilobed. Tarsomeres 1 of meso-metalegs elongated, longer than tarsomeres 2+3 combined, claws appendiculate. ***Abdomen*** (Figs 7B, 9). Margin of fifth sternite convex.

**Male.** The specimens examined are similar in colour and sculpturing to the female. Body smaller, length: 3.9 (0.21); elytral base width: 1.5 (0.06); maximum elytral width: 1.7 (0.10) (*n* = 8). Similar to female in colour and morphology. Abdomen with margin of fifth sternite truncated (Figs 7E, 9B).

##### Description of genitalia

**in specimens from Argentina. *Female genitalia*.** Sternite lamina 8 (Fig. 10E) subtriangular, lateral margins irregular, distal third of lamina sclerotized, tignum thin ~ 2.5 × lamina length, basal portion widened, broad. Vaginal palpi (Fig. 10F) short, conical, slightly divergent. Spermatheca (Fig. 10G) V-shaped, receptacle and pump sclerotised, receptacle oval, distal half narrow, subcylindrical, shorter than receptacle, with a very small appendix at apex. Basal portion of spermathecal duct short, cylindrical, distal part coiled.

***Male genitalia*** (Fig. 10B–D). Aedeagus with median lobe sclerotised, symmetrical, in dorsal view widened from middle of aedeagus, tapering towards end, apex ventrally inclined with small projection, in lateral view slightly curved. Dorsal depression long, ~ 1/2 of median lobe, margins with denticles, one median lamella and two lateral, median lamella triangular, broad and long, ~ 5 × the width of laterals. Ventral depression ~ ¾ of aedeagus. Internal sac with broad U-shaped sclerite, lateral arms broader at the base, connected by a slightly sclerotised area. Tegmen Y-shaped, manubrium and arms of similar length.

##### Variations.

The paralectotypes and other specimens of *L.flavipes* examined are similar to the lectotype specimen; however, there is variation in colouration, punctation, and details of the genitalia. It is necessary to consider the possible colour variations of the type series specimens that were collected before 1859. The colouration variations observed in mature, living specimens are often related to light incidence due to cuticle reflection. For example, the colouration of the Montevideo paralectotype specimen (Fig. 7D) shows variation in the colour of the pronotum that is metallic greenish; the ventral surface of antennomere 1 and the apex of the remaining antennomeres are somewhat darker than in the type, while the elytra are a paler bronze. In the paralectotype from Buenos Aires, the general colour is metallic bronze (Fig. 7F) and the yellow legs are paler. Some specimens collected in Argentina display a darker colour on the antennae and pronotum.

Punctation may vary due to the depth and distance between them. The greatest variation is observed in the punctation near the humeral calli where, in some specimens, is arranged in two rows surrounding the humeral calli. Female genitalia show minor variations. The lamina of sternite 8 is the most variable structure, its shape being highly irregular in some specimens, while in others there may be slight differences in the degree of divergence of the vaginal palpi and development of spermatheca.

##### Geographic distribution.

*Graptoderaflavipes* was originally described from Argentina (Buenos Aires) and Uruguay (Montevideo). Later Bechyné (1954, 1957b, 1959) recorded it in other Argentine provinces (Entre Ríos, Misiones, Corrientes, and Córdoba), Brazilian states (Mato Grosso, Santa Catarina, and Rio Grande do Sul), Bolivia, and Paraguay. Scherer (1960) recorded it for Amazonas (Brazil) and Río Negro province (Argentina). In this work, its distribution is expanded to more localities of the mentioned Argentine provinces and the southern limit of distribution is extended to the province of Neuquén (38°S).

##### Host plants.

*Lysathiaflavipes* was found feeding on Ludwigiagrandiflorasubsp.hexapetala, Ludwigiapeploidessubsp.montevidensis (Onagraceae) and *Myriophyllumaquaticum* (Haloragaceae).

##### Comments.

Bechyné (1954) described two new species, *Alticabohumilae* and *Alticamuriensis*, and included them in a key with *A.flavipes*. He grouped these species, later transferred to the genus *Lysathia*, based on their general copper to purplish colouration, with yellow legs and antennae (Bechyné 1959). *Lysathiaflavipes* and *L.bohumilae* share the tarsomere of the male prolegs being less developed than that of *L.muriensis*. *Lysathiabohumilae* (cited for Argentina: Buenos Aires, Entre Ríos; Paraguay; and Brazil: Rio Grande do Sul) is a robust species, 4.5–5.5 mm, dark brown in colour, with very fine body punctation that decreases in the apical region of the elytra. *Lysathiamuriensis*, cited for Rio de Janeiro (Brazil), differs from the other two species by the the tarsomere 1 of the male prolegs that is as wide as the base of the tibia. Other differentiating characters include a yellow general body colour with pale brown lateral areas on the elytra, and dense pubescence on the apical part of the elytra.

Bechyné included a new species, *L.louella*, in the “*flavipes* group” (Bechyné 1954), cited for Argentina (Tucumán and Salta), which so far does not share its distribution with the species mentioned above. Similar in size to *L.flavipes*, it differs from the other species by the structure of the elytra and its general brown colour with steel blue reflections and reddish antennae. Later, Bechyné (1957a) described *L.viedma* and *L.patagonica* for southern Argentina, relating them to the *Lysathia* species reported for Chile due to their dark (sombre) colouration in legs and antennae (sometimes reddish), in contrast to those of *L.flavipes* group. In (1959), Bechyné described *L.chaparensis*, *L.arapata*, and *L.hygrobia* for Bolivia, associating these three species with the “*flavipes* group”. The broad development of the tarsomere 1 of the male prolegs relates these species to *L.muriensis*.

Much later, Medvedev (2001) described *Lysathiasiolii* from specimens collected on *Ludwigianatans* for the Pantanal region (alluvial plain shared by Mato Grosso do Sul, Mato Grosso, part of Bolivia and Alto Paraguay). He placed this species in the *flavipes* group, close to *L.bohumilae*, *L.flavipes*, and *L.hygrobia* (Bechyné 1959) from Bolivia because of its general bronze colouration and piceous antennae with dark ochre inner part. However, *L.siolii* differs by the dense and deep punctation of the elytra and a well-developed ventral ridge of the median lobe of aedeagus.

#### ﻿ Lysathia cilliersae

Taxon classificationAnimaliaColeopteraChrysomelidae

Cabrera, sp. nov.

A9CAFB29-0F50-583C-A5D9-9893917CAB19

https://zoobank.org/4557971C-2EBD-4581-AFFE-D09BACCEE39A

**Figure 11.**
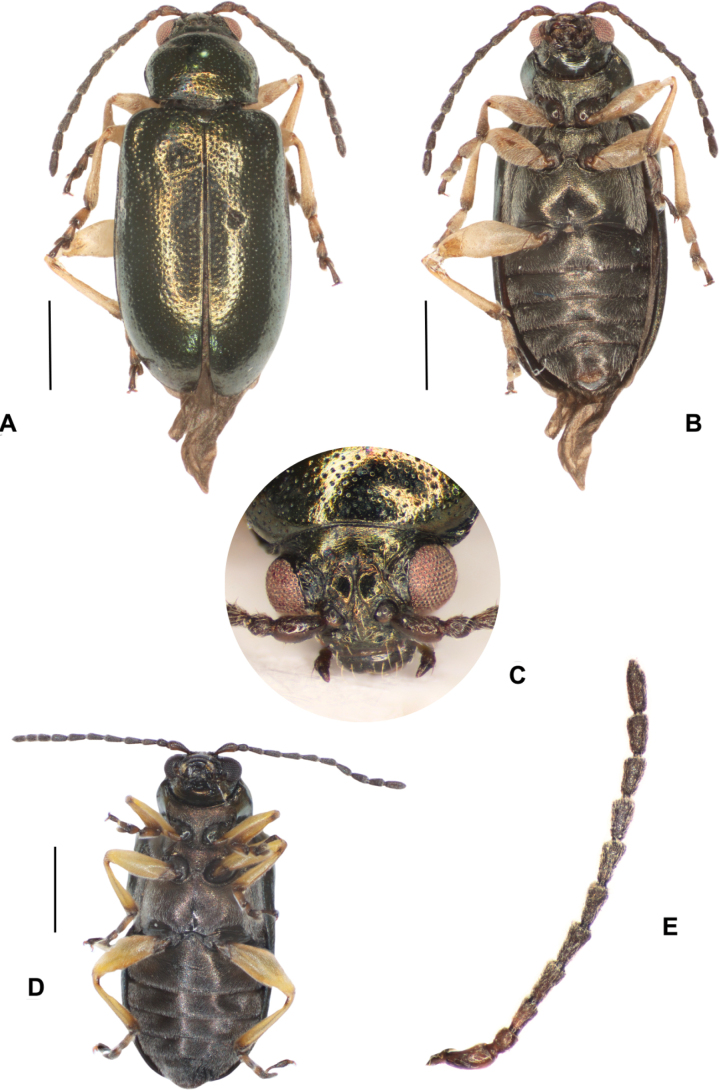
*Lysathiacilliersae* sp. nov. holotype. male (code MA_T_01) **A** dorsal view **B** ventral **C** head. The eye colour of this specimen is altered by the treatment for DNA extraction **D** ventral view of fresh specimen **E** antenna. Scale bars: 1 mm.

**Figure 12.**
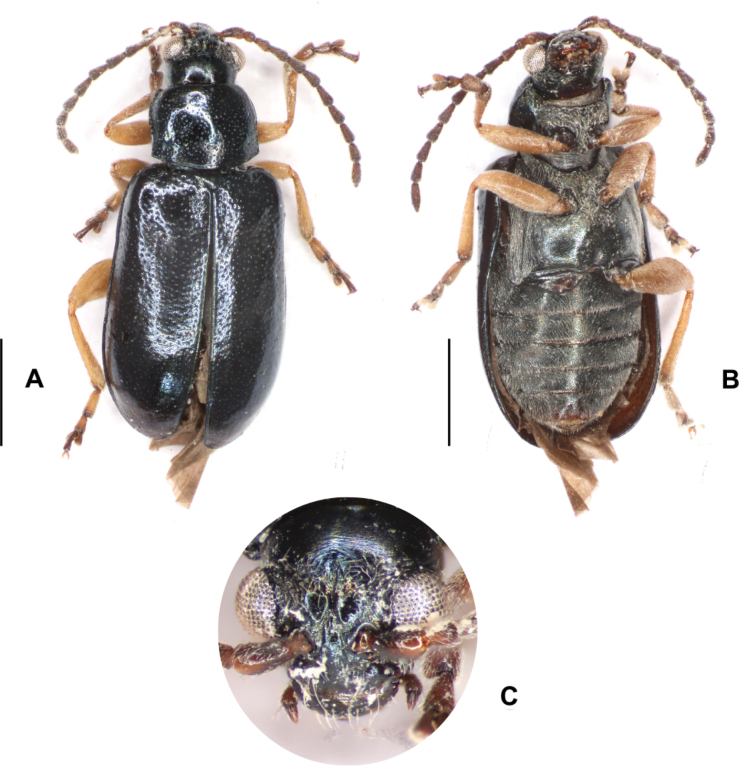
*Lysathiacilliersae* sp. nov. male (code MA_T_03). Colour variability in habitus **A** piceous back with silver-blue reflections **B** ventral, piceous colour **C** head. Scale bars: 1 mm.

**Figure 13.**
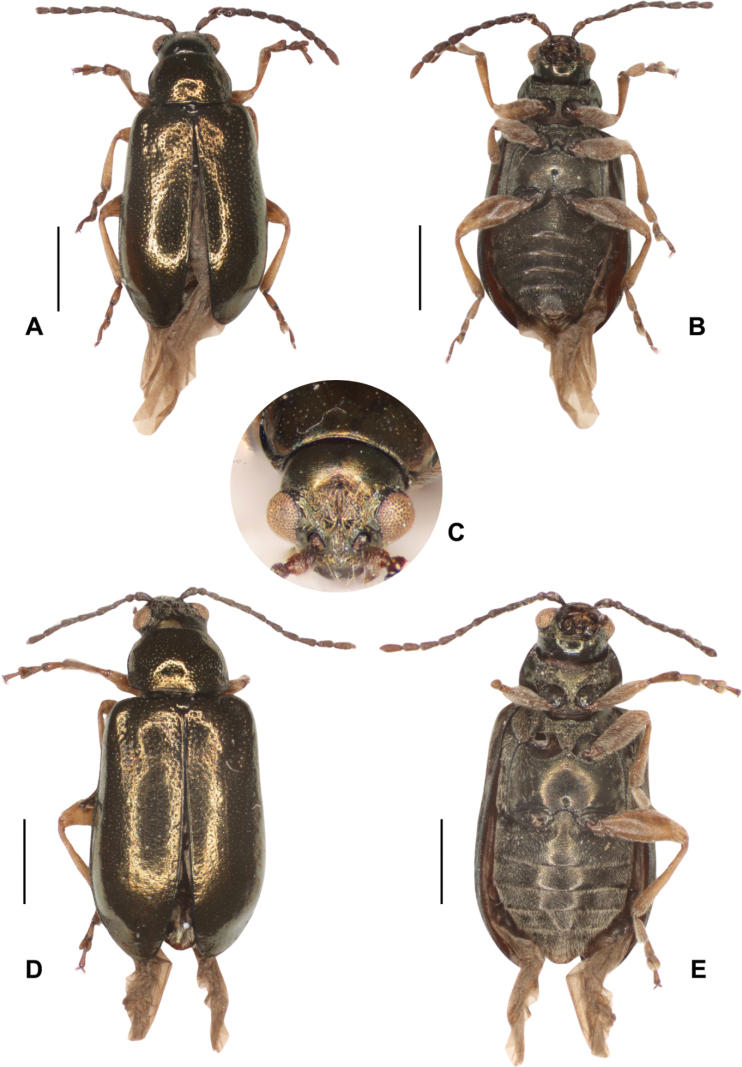
*Lysathiacilliersae* sp. nov. Specimens from mass rearing at the Centre for Biological Control (CBC), Makhanda, South Africa. Male specimen (code MA_I_02) **A** dorsal view **B** ventral view **C** head. Female specimen (code MA_I_01) **D** dorsal view **E** ventral view. Scale bars: 1 mm.

**Figure 14.**
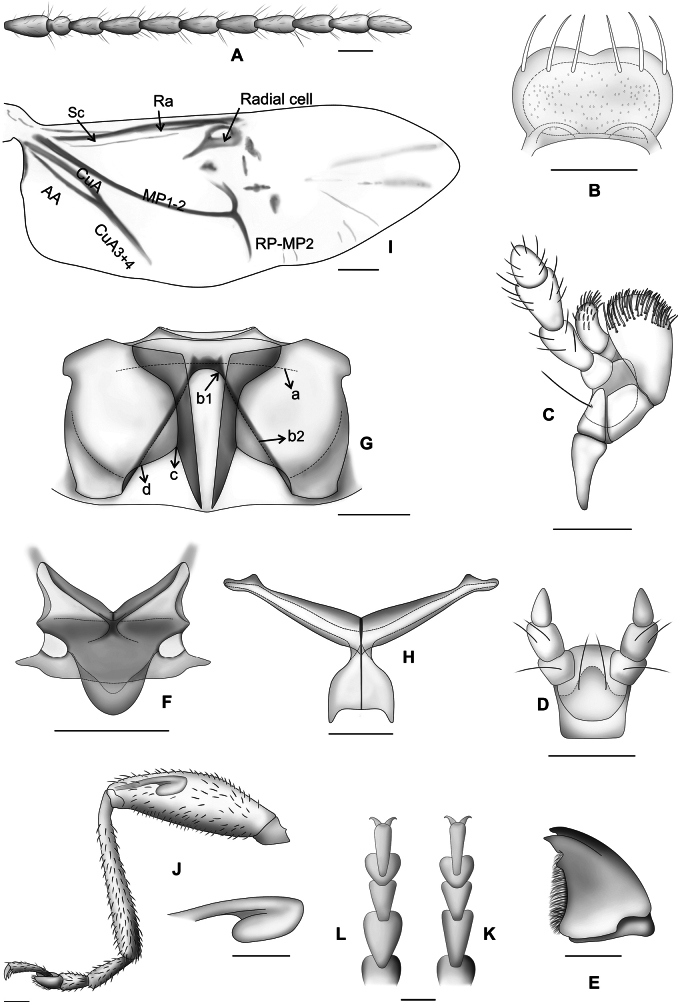
*Lysathiacilliersae* sp. nov. **A** antenna **B** labrum, dorsal view **C** maxilla, ventral view **D** labium, ventral view **E** mandible, external view **F** scutellum **G** metanotum **H** metendosterite, dorsal view **I** hind wing **J** leg and jumping organ of the metafemur **K** tarsitus of female proleg **L** tarsitus of male proleg. Abbreviations: **a**, metanotal bridge; **b1**, metanotal bridge b1; **b2**, metanotal bridge b2; **c**, metanotal bridge c; **d**, metanotal bridge d; **AA**, anal vein AA. **CuA**, cubital anal vein; **CuA 3+4** cubital anal vein 3+4; **MP 1+2**, posterior medial vein 1+2; **RA**, radial vein; **RAc**, radial cell; **SC**, subcostal vein. Illustrations Dr. Julia Rouaux. Scale bars: 0.1 mm.

**Figure 15.**
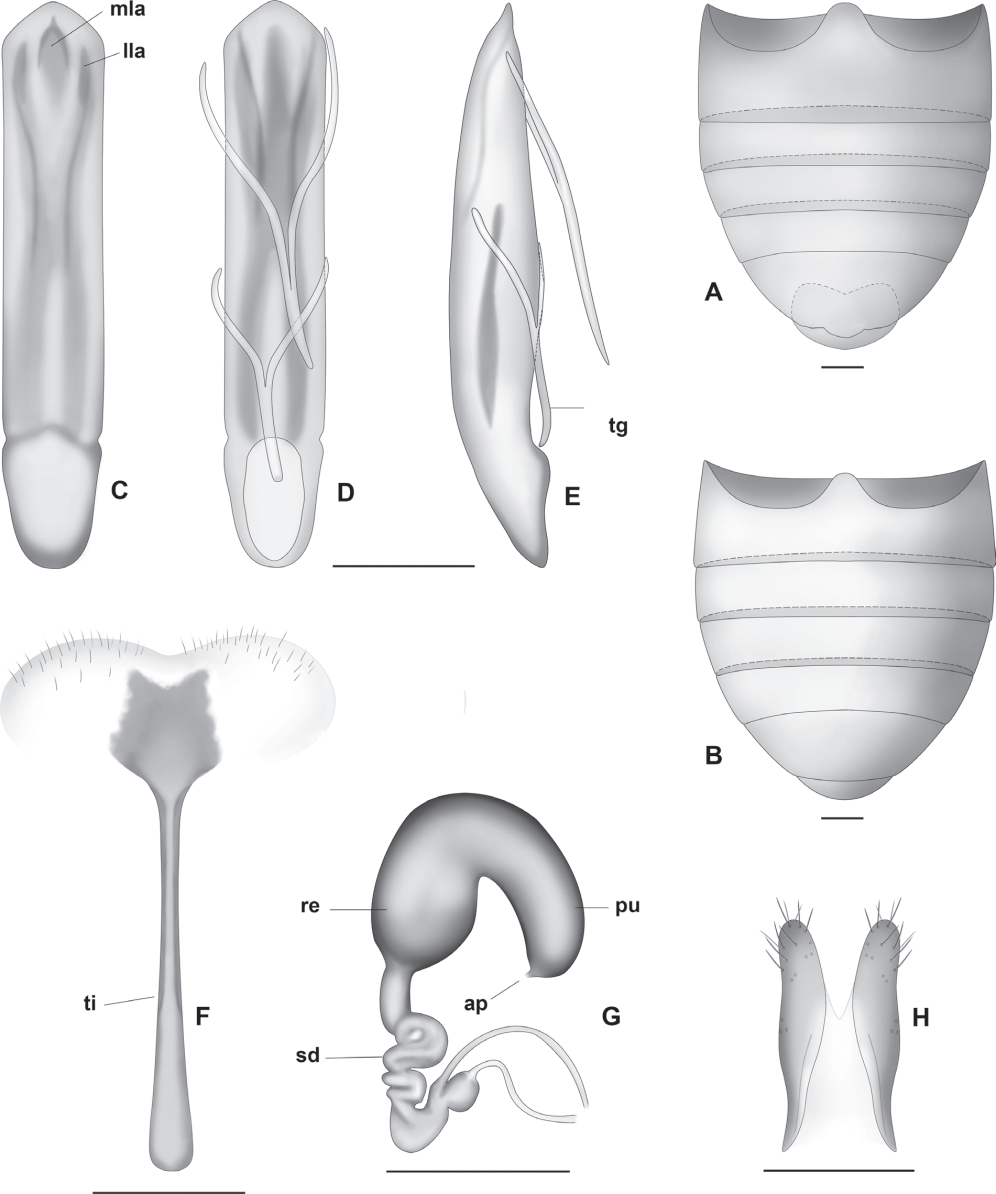
*Lysathiacilliersae* sp. nov. **A, B** abdomen, ventral view **A** male **B** female **C–E** median lobe of aedeagus **C** dorsal view **D** ventral view **E** lateral view **F** sternite 8 **G** spermatheca **H** vaginal palpi. Abbreviations: **ap**: appendix, **lla**: lateral lamella, **mla**: median lamella, **pu**: pump, **re**: receptacle, **sp**: spermathecal duct, **tg**: tegmen, **ti**: tignum. Illustrations Dr. Julia Rouaux. Scale bars 0.1 mm.

Figs 11 , 12 , 13 , 14 , 15

##### Type material.

***Holotype***: Argentina – **Misiones prov.** • 1♂ pinned; Domingo Savio (Ruta 6, 32 km E de Gob. Roca); -27.25561, -55.29895; 15 Nov 2022; A. Faltlhauser leg.; on *Myriophyllumaquaticum*, chemically treated for DNA extraction, left hind leg missing; GenBank: PQ558487, PQ621630; Collectors code: LCMAT01 (45); MLP. ***Paratypes***: Argentina – **Misiones prov.** • 1♀; Domingo Savio (Ruta 6, 32 km E de Gob. Roca); -27.25561, -55.29895; 7 Jan 2024; A. Faltlhauser leg.; blue, on *Myriophyllumaquaticum*, chemically treated for DNA extraction; GenBank: PQ556199; Collectors code (C.c.): LCMAT04 (50); MLP • 1♀,1♂; same locality as for preceding; 7 Jan 2024; A. Faltlhauser leg.; on *Myriophyllumaquaticum*; MLP • 1♂ with genitalia in a separate microvial; same locality as preceding; 7 Jan 2024; A. Faltlhauser leg.; blue, on *Myriophyllumaquaticum*, chemically treated for DNA extraction; GenBank: PQ556198; C.c.: LCMAT03 (49); SANC. • 1♀ with genitalia in a separate microvial; same locality as preceding; 7 Jan 2024; A. Faltlhauser leg.; bronze, on *Myriophyllumaquaticum*, chemically treated for DNA extraction; C.c.: LCMAT06 (52); SANC. • 1♂; same locality as preceding; 15 Nov 2022; A. Faltlhauser leg.; bronze, on *Myriophyllumaquaticum*, chemically treated for DNA extraction; GenBank: PQ558488, PQ621631; C.c.: LCMAT02 (46); FUEDEI • 1♂; same locality as preceding; 7 Jan 2024; A. Faltlhauser leg.; bronze, on *Myriophyllumaquaticum*, chemically treated for DNA extraction; GenBank: PQ558489; C.c.: LCMAT05 (51); FUEDEI. – **Entre Ríos prov.** • 1♂; Calabacillas, cañada Venancio, (Rt.14, km 236); -31.5444953, -58.1873180; 8 Jan 2024; A. Faltlhauser leg.; blue, on *Myriophyllumaquaticum*; C.c.: LCMAB01 (53); MLP. South Africa – **Eastern Cape.** • 1♀; Makhanda, Waainek Mass Rearing Facility (CBC); -22.438539, -44.531849; 25 Nov 2019; A. Faltlhauser leg.; bronze, couple A, on *Myriophyllumaquaticum*; GenBank: PQ558476, PQ621621; C.c.: LCMAI01 (1); MLP • 1♂; same locality and date as for preceding; A. Faltlhauser leg.; bronze, couple A, on *Myriophyllumaquaticum*, chemically treated for DNA extraction; GenBank: PQ558477, PQ621622; C.c.: LCMAI02 (2); MLP • 1♀; same locality and date as for preceding; A. Faltlhauser leg.; couple B, on *Myriophyllumaquaticum*, chemically treated for DNA extraction; GenBank: PQ558478, PQ621623; C.c.: LCMAI03 (3); MLP • 1♂; same locality and date as for preceding; A. Faltlhauser leg.; bronze, couple B, on *Myriophyllumaquaticum*, chemically treated for DNA extraction; GenBank: PQ558479; C.c.: LCMAI004 (4); MLP • 1♀; same locality and date as for preceding; A. Faltlhauser leg.; bronze, couple D; on *Myriophyllumaquaticum*, chemically treated for DNA extraction; GenBank: PQ558482, PQ621625; C.c.: LCMAI007 (26); SANC • 1♂; same locality and date as for preceding; A. Faltlhauser leg.; bronze, couple D; on *Myriophyllumaquaticum*, chemically treated for DNA extraction; GenBank: PQ558483, PQ621626; C.c.: LCMAI08 (27); SANC • 1♀; same locality and date as for preceding; A. Faltlhauser leg.; bronze, couple C; on *Myriophyllumaquaticum*, chemically treated for DNA extraction; GenBank: PQ558480, PQ621624; C.c.: LCMAI05 (5); FUEDEI • 1♂; same locality and date as for preceding; A. Faltlhauser leg.; bronze, couple C; on *Myriophyllumaquaticum*, chemically treated for DNA extraction; GenBank: PQ558481; C.c.: LCMAI06 (6); FUEDEI • 1♂; same locality and date as for preceding; A. Faltlhauser leg.; bronze, couple E; on *Myriophyllumaquaticum*, chemically treated for DNA extraction; GenBank: PQ558484, PQ621627; C.c.: LCMAI09 (28); SLP-art • 1♀ same locality and date as for preceding; A. Faltlhauser leg.; bronze, couple E; on *Myriophyllumaquaticum*, chemically treated for DNA extraction; GenBank: PQ558485, PQ621628; C.c.: LCMAI10 (29); SLP-art.

##### Other material examined.

Argentina • 10♂, 10♀; Domingo Savio, Misiones (Ruta 6, 32 km E de Gob. Roca); -27.25561, -55.29895; 7 Jan 2024; A. Faltlhauser leg; on *Myriophyllumaquaticum*; FUEDEI, SLP-art. South Africa • 10♂, 10♀; Waainek Mass Rearing Facility (CBC), Makhanda, Eastern Cape; 25 Nov 2020; A. Faltlhauser leg; on *Myriophyllumaquaticum*; FUEDEI, SANC.

##### Diagnosis.

This species can be identified by the following characteristics: general colouration piceous or dark brown with golden to green reflections (Fig. 11), dark bronze reflections (Fig. 13), or black with silver to bluish reflections (Fig. 12); Antenna piceous in colour, antennomere 2 globose. Antebasal ridge of pronotum very weakly impressed. Lamina of sternite 8 with lateral margins irregular; coxites long, thin, divergent; baculi weakly developed. Median lobe widened and tapering slightly towards the apex, margins of dorsal depression without denticules. Distal end of tibias and tarsomeres brown.

##### Description of the holotype male

**(Fig. 11).** Length, 3.9 (0.13); elytra base width: 1.7 (0.03); maximum elytral width: 1.9 (0.07); (*n* = 5). Dorsal habitus piceous, or dark brown, with golden to green reflections. Ventral, dark brown (Fig. 11A, B). Antennae dark brown, basal 1/2 of antennomere 1 and inner margin of antennomere 1–3 yellowish. Distal portion of mandibles and labrum tinged with yellow. Legs yellowish white, trochanters tinged with chestnut near coxae, apex of femora and tibiae tinged with brown; tarsomeres 1 and 2 yellowish, with distal portion dark brown, tarsomere 3 brown (Fig. 11D).

***Head*** (Fig. 11C) Vertex glabrous, more or less convex in lateral view; coarse punctures situated between posterior margin of antennal calli, inner margin of eyes and at least a pair of large supraorbital punctures; two or three pairs of long supraorbital setae; postantennal calli barely raised, trapezoidal, touching in the inner posterior angles; supracallinal sulcus strongly impressed; antennal sockets closed to anterior margin of eyes, distance between them slightly greater than transverse diameter of antennal sockets, two or three short setae below antennal sockets and two long setae near base of clypeus. Frontal ridge, moderately convex, anterofrontal ridge slightly lower than frontal ridge in lateral view, with row of small setae on each side. Antennae (Figs 11E, 14A) inserted below midline of eyes, extending 1/2 length of elytra; antennomere 2 short, globose, ~ 2 × the length of 3, antennomere 3 elongate, a little shorter than 4, antennomeres 4–7 elongate, similar in length, antennomeres 8–10 rather shorter, antennomere 11 apically acuminate. Antennomeres 1–3 scarcely setose, antennomeres 4–11 densely setose throughout, all antennomeres with erect, sparse setae at apex. Eyes convex, inner margin straight. Labrum (Fig. 14B) rectangular, distal margin slightly emarginated, with a row of six long setae near distal margin. Mandibles (Fig. 14E) symmetrical, 4-toothed apically, teeth 2–3 visible on external face, teeth 2–3 acute, subequal, tooth 4, short, acute. Maxillae (Fig. 14C) cardo apically broadened; galea subconical and lacinia cylindrical, both with fringelike pilosity, scarcer and shorter in galea. Maxillary palpi 4-segmented, palpomere 1 quadrangular, short; palpomeres 2 subcylindrical, equal, palpomere 3 subconical, rather shorter than 2, palpomere 4 subconical short, with wide base, tapering apically. Labium (Fig. 14D) with two setae between bases of palps, ligula rounded at apex; labial palp 3-segmented, palpomere 1 rectangular; palpomere 2 subconical, > 2 × longer than 1; palpomere 3 subconical with narrow base.

***Thorax*.** Pronotum slightly convex, shiny, finely punctate (1.2 wide × 0.8 mm long, *n* = 5), maximum width near the middle of the pronotum; antebasal sulcus very weakly impressed, scarcely visible, not reaching lateral margins; anterior lateral angles rounded and posterior lateral angles acuminate with a long, thin seta on each one. Surface with scattered punctures, more abundant at the base. Prosternum convex; finely pubescent, prosternal process narrow, rounded at apex, extending beyond posterior margin of procoxae, procoxal cavities oblong, open behind. Mesosternum densely microsculptured and pubescent, mesosternal process broad and short; mesocoxal cavities rounded inserted on posterior margin. Scutellum (Fig. 14F) triangular, smooth, rounded at apex. Metanotum (Fig. 4G) transverse, wider than long; metanotal ridge *d* intersecting *c* posteriorly to midpoint of *c.* Metasternum transverse, slightly concave centrally, with slightly pronounced bidentate projection between metacoxae; metacoxal cavities transverse, inserted at posterior margin. Metasternum usually broader than long, central area finely striate, densely pubescent in lateral areas, metasternal longitudinal suture visible near base of metacoxae, metacoxal cavities transverse inserted on posterior margin. Meta-endosternite (Fig. 14H) T-shaped, stalk wider at base, shorter than lateral arms, meso- and metafurcal tendons well developed, inserted near apex of lateral arms. Elytra oval, convex, slightly wider than pronotum, greatest width near the posterior one-third of elytra; humeral calli rounded, strongly produced. Surface densely, uniformly punctate; punctures somewhat coarser than on pronotum, more finely punctate at apex; discal punctures partly aligned in short rows, close sutura elytral. Hind wings (Fig. 14I) with veins RA, MP well sclerotised whereas veins SC, CuA, RP-MP2, and AA scarcely sclerotised. Vein SC connected to RA less than half its length, radial cell darkly pigmented, elongate, subtriangular; AA connected to CuA3+4 more than one-half the distance from origin of CuA; cubital anal cell closed, elongate. Legs with trochanters triangular, pro-and mesofemora almost parallel-sided, metafemora larger, dorsal margin evenly convex, ventral margin almost straight; apical margins of all tibiae with short spurs; trochanters, femora and tibiae of all legs sparsely setose; femora of metalegs densely setose ventrolaterally. Hind legs with metafemoral jumping organ (Fig. 14J). Tarsomere 1 of prolegs wider in male than in female (Fig. 14L, K), suboval, broad, shorter than tarsomeres 2+3 together, tarsomere 2 triangular, tarsomere 3 deeply bilobed; tarsomere 1 of meso- and metalegs elongate, longer than tarsomeres 2+3 together; tarsal claws appendiculate.

***Abdomen*.** (Fig. 15A) apex of ventrite 5 with a middle truncate lobe on posterior margin. Genitalia. Median lobe (Fig. 15C, D) thin, sclerotised, symmetrical, widened in dorsal view, tapering slightly toward the apex, scarcely deflexed, apically with a small projection scarcely visible in dorsal view, evenly curved in lateral view, slightly constricted at the basal ~ ¼. Dorsal depression usually long, ~ ¾ length of the median lobe, two lateral and a median lamella, median lamella wide at base, longer than laterals; ventral depression long, almost 3/4 its length margins slightly elevated. Internal sac with a large U-shaped sclerite, each lateral arms long, strongly sclerotised, anterior connecting portion weakly sclerotised. Tegmen Y-shaped, manubrium a little shorter than arms.

**Female.** The specimens studied are similar in size to males, length: 3.9 (0.18); elytral base width: 1.6 (0.07); maximum elytral width: 1.8 (0.19); (*n* = 7). Similar to males in colour and body sculpture. Tarsomere 1 of prolegs (Fig. 14K) triangular, narrower than in males. Abdomen (Fig. 15B) broader than in males, apical region of sternite 5 slightly convex. Genitalia: lamina of sternite 8 subtrapezoidal (Fig. 15F), irregular margins, distal half of lamina sclerotised, tignum very thin, little > 3 × the length of lamina, basal part slightly enlarged. Vaginal palpi (Fig. 15H) long, thin and divergent. Spermatheca (Fig. 15G) V-shaped, receptacle and pump sclerotised, receptacle rounded, pump subcylindrical, narrow, and somewhat longer than receptacle, with a very small appendix at apex. Basal portion of spermathecal duct cylindrical, narrow and long, distal part coiled.

##### Variations.

**Figure 16.**
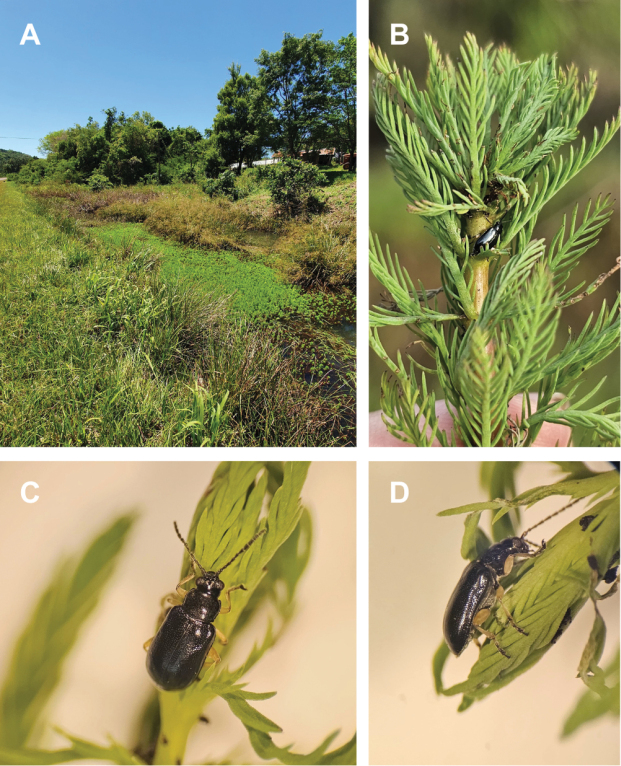
**A** type locality where *Lysathiacilliersae* sp. nov. was collected on *Myriophyllumaquaticum* (Misiones, Rt. prov. 6, Domingo Savio) **B** field-collected specimen of *L.cilliersae*. Live specimens of *L.cilliersae* sp. nov. **C** dorsal view **D** lateral view.

In *Lysathiacilliersae* sp. nov. differences are observed among specimens in body colouration and punctation. The body colour primarily varies in the tone of reflections, ranging from bronze-gold, bronze-green, to metallic blue (Figs 11–13). A distinctive feature observed in the specimens from Misiones is the leg colouration: a very pale yellow that contrasts with the darker areas of the coxae, trochanters, distal part of the tibiae, and tarsomeres (Figs 11D, 16C, D). However, in one female, the legs appeared a somewhat “dirtier” and darker yellow, similar to most specimens from the mass rearing in CBC, South Africa (Fig. 13).

Variation in the punctation of the pronotum and elytra was noted in specimens of both sexes from South Africa and Misiones, making it difficult to establish a definitive punctation pattern. The variation observed in the pronotum refers to some specimens having deeper and denser punctation in the basal region. In some specimens, the elytral punctation is slightly deeper than in the pronotum. In all specimens, the punctation of the elytra decreases towards the distal portion. However, differences are evident in the basal region of the elytra: in some specimens, the punctation is faint and disorganised, while in others, one or two rows of less pronounced punctures are aligned parallel to the elytral suture in the upper part. The rounded and prominent humeri are bare, and in some specimens, there is a more elevated area near the humeri, where the punctures are fainter and scattered. In contrast, in others, the punctures are more robust and arranged in a row parallel to the humeri.

##### Etymology.

The name of this species is dedicated to Dr. Carina J. Cilliers, ARC, Plant Protection Research Institute, South Africa, who studied and released this species as a biological control agent of *Myriophyllumaquaticum*.

##### Geographic distribution

**(Fig. 2).***Lysathiacilliersae* sp. nov. is currently known from Argentina, near the locality of Domingo Savio (-27.25561, -55.29895) in Misiones province. It was also recorded further south in Calabacillas, Entre Ríos (-31.5444953, -58.1873180). Additionally, genetic studies revealed that the specimens reared in South Africa, originally sourced from Penedo, Rio de Janeiro, Brazil, share the same haplotype as those from Misiones. Thus, it is inferred that the distribution of *L.cilliersae* sp. nov. extends between these two localities.

##### Biological notes.

Laboratory crosses were carried out between specimens of *Lysathiacilliersae* sp. nov. exhibiting different colouration to observe whether they produced offspring and of which colouration. Naive females from Misiones and Calabacillas, Entre Ríos (locality not included in the genetic studies) were used. In a cross between a black female with silver-blue reflections from Misiones and a male with gold-green reflections from Entre Ríos, offspring of both colours were obtained: one male and three black females, and one male and three greenish gold females. In another cross between a golden-green pair from Misiones, 27 offspring were obtained, all golden-green. Finally, a black pair produced 29 black and seven green-gold offspring. Although these crosses are preliminary, it can be concluded that the different colouration does not indicate the existence of different biological species.

##### Comments.

The small size of *L.cilliersae* sp. nov. as well as its colouration and punctation are similar to species in the *L.flavipes* group. Nevertheless, *L.cilliersae* sp. nov. can be distinguished from the *L.flavipes* group by their dark brown to black antennae, the globose antennomere 2, and the barely perceptible antebasal ridge of the pronotum, whereas in some species of the *flavipes* group, this ridge is more pronounced. Additionally, the leg colouration in some specimens is very pale, almost whitish yellow. The genital structures provide useful characters for species identification; however, more research is required for species within *Lysathia*, except for the aedeagus of *L.siolii* and the male and female genitalia of *L.flavipes*. Regarding the female genitalia, the shape of sternite 8 varies in both species, with the lamina having irregular margins. Some differences are also observed in the vaginal palpi and spermatheca. Although the degree of divergence in the inner margin of the vaginal palpi is variable, the baculi in females of *L.cilliersae* sp. nov. are barely perceptible, whereas in *L.flavipes*, they are heavily sclerotised. In the spermatheca of *L.cilliersae* sp. nov., the distal part reaches the base of the receptacle, while in *L.flavipes*, the receptacle is rounded and the distal part does not reach the base. In males, one of the most notable characteristics is the absence of a denticle on the margin of the dorsal depression and the development of the median sulcus on the ventral surface of the median lobe.

## ﻿Discussion

In this study, an integrative taxonomic approach was applied combining genetic and morphological analysis to expand the description of *Lysathiaflavipes* and describe a new species, *Lysathiacilliersae* sp. nov. The research revealed morphological and genetic variation within the *L.flavipes* specimens studied and extended its known distribution in Argentina, providing a more comprehensive description of the species. No evidence was found to suggest that *L.flavipes* represents a species complex associated with its different host plants, *Ludwigiag.* subsp. hexapetala, Ludwigiap.subsp.montevidensis, and *M.aquaticum*. On the other hand, *L.cilliersae* sp. nov. was formally described, further contributing to the understanding of this successful biological control agent against *M.aquaticum* and expanding its range from Brazil to Argentina.

The phylogenetic trees constructed in this study support the taxonomic revision made by Bechyné (1959), which separated *Lysathia* from *Altica*. Distinct and well-supported clades of *Lysathia*, as well as the clear separation from the included *Altica* species, show that the *Lysathia* species evaluated here form a cohesive and monophyletic group. Jenkins et al. (2009) had previously identified *Altica* as genetically separate from *Lysathia* although they only studied a single species (*Lysathia* sp.), likely *L.ludoviciana*. The results obtained here support this generic distinction, which has been questioned in the past by taxonomists due to the “subtle” morphological differences (Scherer 1962) and biological similarities with some species of *Altica* (McGregor et al. 1996; Takizawa 2005).

This genetic study has helped in revealing the genetic variability and potential species differentiation within the genus *Lysathia*. Accurate identification at species level within this genus is particularly challenging due to the significant morphological variability and the limited detail and illustration in the existing descriptions. Although molecular techniques alone do not resolve taxonomic challenges, they facilitate the identification of lineages, the comparison of different populations, and offer additional insights alongside morphological analysis (Jenkins et al. 2009). Such taxonomic difficulties are also observed in the related genus *Altica*, where traits generally considered constant in other alticines, like the antennal calli, oviscapt, and spermatheca, display substantial variation across species (Döberl 2010).

The phylogenetic and morphological analyses conducted in this study revealed that *L.flavipes* is a species with greater morphological and genetic variation than originally assumed. Although the bootstrap value obtained from the RAxML tree for the COI gene was low, the consistency in the positioning of the specimens in the combined COI and 16S gene tree and the improved bootstrap value provided additional support for the monophyly of *L.flavipes*. Based on both analyses, we can consider that, with the exception of two samples, all specimens belong to the same species. However, more data, including incorporating other genetic markers and more specimens, are recommended for further confirmation. For example, in this work the amplification of the nuclear gene Wg was not successful, but future research could benefit from the information this gene provides. The morphological variations observed in the collected specimens do not seem to be indicators of different species. This could be consistent with the significant number of haplotypes observed for *L.flavipes*. Of the 22 sequences analysed, 18 different haplotypes were observed. Although this study did not focus on the species’ phylogeography, the haplotype network suggests that Buenos Aires and Entre Ríos could be a centre of origin with more ancestral haplotypes and that the species may have spread outward from this region. Another interesting result is the confirmation that the specimens collected in Uruguay and evaluated in USA (Reddy et al. 2021a) are, indeed, *L.flavipes* and therefore the studies on its biology and reproductive characteristics can be applied to the general characterisation of the species.

On the other hand, the initial hypothesis that the host plants on which each specimen was found feeding could indicate a possible species complex was not confirmed. No genetic or morphological groupings were observed related to the host plants *Ludwigiag.* subsp. hexapetala or *M.aquaticum*. Nor were there differences among specimens collected in sites where these plants are found in sympatry or allopatry. This, on the one hand, supports that *L.flavipes* is not a suitable candidate as a control agent for *L.g.* subsp. hexapetala, but it also raises several questions regarding the chemical similarities between these two plants, that are not phylogenetically related but to which, according to previous studies, *Lysathiaflavipes* restricts its diet (Cordo and DeLoach 1982).

Of the specimens collected as *L.flavipes*, only two specimens consistently fell outside the species. Specimen MA_F_01 (referred to as *Lysathia* sp. 1 in the phylogenetic trees and haplotype network), from Otamendi, Buenos Aires, appeared in both trees as a sister species of *L.cilliersae* sp. nov. Its differentiation was also evidenced morphologically in its antennae, legs, and female genitalia. As only one specimen was available, a formal description of a new species was not made, although a morphological description and mitochondrial COI and 16S ribosomal genes sequences were provided for future studies (Suppl. material 1: Partial morphological description for *Lysathia* sp.1 (code MA_F_001)). On the other hand, specimen LH_M_01 (*Lysathia* sp. 2) from Gorchs, Buenos Aires, proved inconclusive regarding its phylogenetic placement. Depending on the gene studied, it positioned as a sister species to either *L.flavipes* or *L.ludoviciana*, in both cases with low branch support values. It is likely that, with the inclusion of additional genes and more specimens from both localities, the ambiguities observed here could be resolved.

Although the results suggest dismissing *Lysathiaflavipes* as a potential control agent for *Ludwigiag.* subsp. hexapetala, the research conducted to reach this conclusion yielded valuable information to make the description of a new species, *Lysathiacilliersae* sp. nov. This result is noteworthy not only because it addresses questions regarding the genus *Lysathia* and its host plants, but also contributes to the understanding of the Neotropic biodiversity. Based on genetic and morphological analyses of *L.cilliersae* sp. nov., it was possible to corroborate that the specimens originally collected in Brazil and later exported as biological control agents for *M.aquaticum* in South Africa, are the same species as those found in Domingo Savio, Misiones, Argentina. The cohesion observed in the clades suggests that, despite the geographical separation, the specimens are genetically very similar, to the extent that the same haplotype was found in both countries, supporting their identification as a single species. Morphologically, polymorphisms are observed as in *L.flavipes*. A feature to highlight is the variation in colouration of *L.cilliersae* sp. nov. between both countries. In Argentina, the studied specimens exhibit variability in dorsal colouration, varying from bronze-gold, bronze-green, to metallic blue. However, the South African specimens are homogeneously bronze coloured. This difference may be due to the fact that, although South Africa has successfully maintained a colony of *L.cilliersae* sp. nov. for many years, the population is the result of a single introduction, which can lead to genetic drift, increased inbreeding and bottleneck effects. These reduces not only genetic variability but also potentially the effectiveness of the agent over time. In this context and after thirty years of its first release as a control agent in South Africa (Cilliers 1999a; Cilliers 1999b), the description of *L.cilliersae* sp. nov. is fundamental.

The lack of taxonomic identification of a biocontrol agent can not only delay the development of new biological control program (Maddox et al. 1992; Zucchi 2002), but also hinder those that are already underway. Insect taxonomy, particularly that of herbivores associated with invasive plants that are not considered agricultural weeds, have certain complications. For example, the amount of information on species of non-economic importance is, as expected, much scarce than other species with direct importance to humans (Shimbori et al. 2023). Integrative taxonomy studies are becoming more frequent and the incorporation of molecular diagnoses based on genetic markers are rapidly increasing in importance within taxonomy, especially used as complementary tools to morphological, behavioural or biological studies in general (Marvaldi 2024).

Although this study is not a revision of the genus, the significance of this work lies in the contribution of information within the context of applied entomology. Because taxonomic validation is essential for both taxonomy and biological control, all the genetic information obtained here is correlated with their respective morphological vouchers and are deposited in public data-bases and public collections.

## Supplementary Material

XML Treatment for Lysathia flavipes

XML Treatment for Lysathia cilliersae

## ﻿Additional information

### Author ORCIDs

Ana C. Faltlhauser https://orcid.org/0000-0003-3121-3650

M. Cristina Hernández https://orcid.org/0000-0003-4382-773X

Andrés Sánchez Restrepo https://orcid.org/0000-0001-7514-068X

Martin Hill https://orcid.org/0000-0003-0579-5298

Alejandro J. Sosa https://orcid.org/0000-0002-1680-8712

### Data availability

All of the data that support the findings of this study are available in the main text or Supplementary Information.

## Supplementary materials

Supplementary material 1 Supplementary file Data type docx Explanation note This file contains genetic protocols for PCR extraction, Lysathia species geographical information of ocurrences, gentic sequences codes, phylogenetic trees and preliminar morphological description for undescribed species. https://binary.pensoft.net/file/1250676This dataset is made available under the Open Database License (http://opendatacommons.org/licenses/odbl/1.0/). The Open Database License (ODbL) is a license agreement intended to allow users to freely share, modify, and use this Dataset while maintaining this same freedom for others, provided that the original source and author(s) are credited.Ana C. Faltlhauser, Nora Cabrera, M. Cristina Hernández, Andrés Sánchez Restrepo, Martin Hill, Alejandro J. Sosa
