# Supplementary material for: ﻿Lysathiaflavipes and Lysathiacilliersae Cabrera sp. nov. (Coleoptera, Chrysomelidae): genetic and morphological unravelling of biocontrol agents for two invasive aquatic plants
Source: Zookeys. 2025 Feb 13;1228:11–52. doi: 10.3897/zookeys.1228.138773 (PMC11843172; doi:10.3897/zookeys.1228.138773)
Supplement: Supplementary material 1 — Supplementary file [file zookeys-1228-011_article-138773__-s001.docx]

**SUPPLEMENTARY FILE**

***Lysathia flavipes* and *Lysathia* sp. nov. (Chrysomelidae): Molecular and morphological unraveling of biocontrol agents for *Ludwigia grandiflora* subsp. *hexapetala* and *Myriophyllum aquaticum.***

Faltlhauser Ana C.^1,2^, Cabrera Nora^3^, Hernández M. Cristina^1^, Sánchez-Restrepo Andrés^1^, Hill Martin^4^ & Sosa Alejandro J.^1,2^

**S.1 - PCR protocols -**

PCR reactions were carried out in a 25 μl volume reaction containing: 4 μl genomic DNA extract, 0.6 μl of each primer, 1.5 μl MgCl_2_, 2.5 μl dNTPs, 0.2 μl Taq polymerase, 2.5 μl PCR Buffer and 16.1 μl of distilled water. Thermal cycling conditions were as follows: denaturation at 95°C for 3 min, then 40 cycles of denaturation at 94°C for 1 min, annealing at 51°C for 1 min, and extension at 72°C for 2 min, followed by a final extension at 72°C for 10 min. PCR products were visualized on a 1% agarose gel stained with GelRed (Biotium, Hayward, CA, USA) and 15 μl of PCR product were purified using a mixture of 0.5μl (10u) FastAP (Thermosensitive Alkaline Phosphatase), 1 μl (1u) ExoI (exonuclease I) incubated at 37°C for 15 min, stopping the reaction by heating the mixture at 85°C for 15 min. Both strands of each fragment were sequenced using Sanger technology in Macrogen Inc. sequencing service (South Korea) with the same primers used for PCR amplification.

**S.2 – Species and origin of the sequences from the Phylogenetic Analyses –**

**Table S2.1:** Specimens of *Lysathia* used in molecular analyses with their respective sample codes; (H.) Host plant on which they were collected: (LM) *Ludwigia p*. subsp. *montevidensis*, (LH) *L. g*. subsp. *hexapetala*, and (MA) *Myriophyllum aquaticum*. (Site) Collection site code. (Indiv.) Individual specimen number from the site and host plant. (Insect) Presumed species of *Lysathia*.

| H. | Site | Indiv. | Insect | Host Plant | Locality | Province | Country | Lat | Long | Collection date | COI | 16S |
| --- | --- | --- | --- | --- | --- | --- | --- | --- | --- | --- | --- | --- |
| LM | A | 001 | *L. flavipes* | *L. p. montevidensis* | Villa Ventana | Buenos Aires | Argentina | -38.06977 | -61.92542 | 28Feb2021 |  |  |
| LM | A | 002 | *L. flavipes* | *L. p. montevidensis* | Villa Ventana | Buenos Aires | Argentina | -38.06977 | -61.92542 | 28Feb2021 | PQ558504 |  |
| LH | B | 001 | *L. flavipes* | *L. g. hexapetala* | Calabacillas | Entre Ríos | Argentina | -31.54151 | -58.18973 | 11Jan2022 | PQ558491 |  |
| LH | B | 002 | *L. flavipes* | *L. g. hexapetala* | Calabacillas | Entre Ríos | Argentina | -31.54152 | -58.18974 | 11Jan2022 | PQ558492 | PQ621632 |
| MA | B | 001 | *L. flavipes* | *M. aquaticum* | Calabacillas | Entre Ríos | Argentina | -31.54153 | -58.18975 | 11Jan2022 | PQ558507 |  |
| MA | B | 002 | *L. flavipes* | *M. aquaticum* | Calabacillas | Entre Ríos | Argentina | -31.54154 | -58.18976 | 11Jan2022 | PQ558508 | PQ621637 |
| MA | B | 003 | *L. flavipes* | *M. aquaticum* | Calabacillas | Entre Ríos | Argentina | -31.54155 | -58.18977 | 11Jan2022 | PQ558509 | PQ621638 |
| MA | B | 001 | *L. cilliersae* | *M. aquaticum* | Calabacillas | Entre Ríos | Argentina | -31.54449 | -58.18731 | 11Jan2022 |  |  |
| MA | F | 001 | *L. flavipes* | *M. aquaticum* | Otamendi | Buenos Aires | Argentina | -34.09885 | -58.79671 | 26Jan2021 | PQ558510 | PQ621639 |
| LH | H | 001 | *L. flavipes* | *L. g. hexapetala* | Punta del Diablo | Rocha | Uruguay | -34.01305 | -53.59830 | 10Mar2019 |  |  |
| LH | H | 002 | *L. flavipes* | *L. g. hexapetala* | Punta del Diablo | Rocha | Uruguay | -34.01305 | -53.59830 | 10Mar2019 |  |  |
| LH | H | 003 | *L. flavipes* | *L. g. hexapetala* | Punta del Diablo | Rocha | Uruguay | -34.01305 | -53.59830 | 10Mar2019 |  |  |
| LH | H | 004 | *L. flavipes* | *L. g. hexapetala* | Punta del Diablo | Rocha | Uruguay | -34.01305 | -53.59830 | 10Mar2019 |  |  |
| LH | H | 005 | *L. flavipes* | *L. g. hexapetala* | Punta del Diablo | Rocha | Uruguay | -34.01305 | -53.59830 | 10Mar2019 |  |  |
| LH | H | 006 | *L. flavipes* | *L. g. hexapetala* | Punta del Diablo | Rocha | Uruguay | -34.01305 | -53.59830 | 10Mar2019 |  |  |
| LH | H | 007 | *L. flavipes* | *L. g. hexapetala* | Punta del Diablo | Rocha | Uruguay | -34.01305 | -53.59830 | 10Mar2019 |  |  |
| LH | H | 008 | *L. flavipes* | *L. g. hexapetala* | Punta del Diablo | Rocha | Uruguay | -34.01305 | -53.59830 | 10Mar2019 | PQ558493 |  |
| LH | H | 009 | *L. flavipes* | *L. g. hexapetala* | Punta del Diablo | Rocha | Uruguay | -34.01305 | -53.59830 | 10Mar2019 | PQ558494 |  |
| MA | I | 001 | *L.* sp. | *M. aquaticum* | Makhanda/  Penedo | W. Cape/Río de Janeiro | Sudáfrica/  Brasil | -33.32004 | 26.50776 | 25Nov2019 | PQ558476 | PQ621621 |
| MA | I | 002 | *L.* sp. | *M. aquaticum* | Makhanda/  Penedo | W. Cape/Río de Janeiro | Sudáfrica/  Brasil | -33.32004 | 26.50776 | 25Nov2019 | PQ558477 | PQ621622 |
| MA | I | 003 | *L.* sp. | *M. aquaticum* | Makhanda/  Penedo | W. Cape/Río de Janeiro | Sudáfrica/  Brasil | -33.32004 | 26.50776 | 25Nov2019 | PQ558478 | PQ621623 |
| MA | I | 004 | *L.* sp. | *M. aquaticum* | Makhanda/  Penedo | W. Cape/Río de Janeiro | Sudáfrica/  Brasil | -33.32004 | 26.50776 | 25Nov2019 | PQ558479 |  |
| MA | I | 005 | *L.* sp. | *M. aquaticum* | Makhanda/  Penedo | W. Cape/Río de Janeiro | Sudáfrica/  Brasil | -33.32004 | 26.50776 | 25Nov2019 | PQ558480 | PQ621624 |
| MA | I | 006 | *L.* sp. | *M. aquaticum* | Makhanda/  Penedo | W. Cape/Río de Janeiro | Sudáfrica/  Brasil | -33.32004 | 26.50776 | 25Nov2019 | PQ558481 |  |
| MA | I | 007 | *L.* sp. | *M. aquaticum* | Makhanda/  Penedo | W. Cape/Río de Janeiro | Sudáfrica/  Brasil | -33.32004 | 26.50776 | 25Nov2019 | PQ558482 | PQ621625 |
| MA | I | 008 | *L.* sp. | *M. aquaticum* | Makhanda/  Penedo | W. Cape/Río de Janeiro | Sudáfrica/  Brasil | -33.32004 | 26.50776 | 25Nov2019 | PQ558483 | PQ621626 |
| MA | I | 009 | *L.* sp. | *M. aquaticum* | Makhanda/  Penedo | W. Cape/Río de Janeiro | Sudáfrica/  Brasil | -33.32004 | 26.50776 | 25Nov2019 | PQ558484 | PQ621627 |
| MA | I | 010 | *L.* sp. | *M. aquaticum* | Makhanda/P  enedo | W. Cape/Río de Janeiro | Sudáfrica/  Brasil | -33.32004 | 26.50776 | 25Nov2019 | PQ558485 | PQ621628 |
| MA | I | 011 | *L.* sp. | *M. aquaticum* | Makhanda/  Penedo | W. Cape/Río de Janeiro | Sudáfrica/  Brasil | -33.32004 | 26.50776 | 25Nov2019 | PQ558486 | PQ621629 |
| MA | J | 001 | *L. ludoviciana* | *Ludwigia sp.* |  | Luisiana | EEUU | 29.669458 | -92.56775 |  | PQ558514 | PQ621641 |
| MA | J | 002 | *L. ludoviciana* | *Ludwigia sp.* |  | Luisiana | EEUU | 29.669458 | -92.56775 |  | PQ558515 | PQ621642 |
| MA | L | 001 | *L. flavipes* | *M. aquaticum* | Las Flores | Buenos Aires | Argentina | -35.95514 | -59.01123 | 06May2022 | PQ558511 | PQ621640 |
| MA | L | 002 | *L. flavipes* | *M. aquaticum* | Las Flores | Buenos Aires | Argentina | -35.95514 | -59.01123 | 06May2022 | PQ558512 |  |
| LH | M | 001 | *L. flavipes* | *L. g. hexapetala* | Gorchs | Buenos Aires | Argentina | -27.49034 | -58.71573 | 26Jan2022 | PQ558495 | PQ621633 |
| MA | N | 001 | *L. flavipes* | *M. aquaticum* | Dique Lujan | Buenos Aires | Argentina | -34.36496 | -58.67688 | 23Sep2021 |  |  |
| LH | P | 001 | *L. flavipes* | *L. g. hexapetala* | La Plata | Buenos Aires | Argentina | -35.00200 | -58.02563 | 21Dec2021 | PQ558496 |  |
| LH | Q | 001 | *L. flavipes* | *L. g. hexapetala* | Plottier | Neuquén | Argentina | -38.96948 | -68.18534 | 21Feb2021 | PQ558497 |  |
| LH | Q | 002 | *L. flavipes* | *L. g. hexapetala* | Plottier | Neuquen | Argentina | -38.96948 | -68.18534 | 21Feb2021 | PQ558498 |  |
| LH | Q | 003 | *L. flavipes* | *L. g. hexapetala* | Plottier | Neuquen | Argentina | -38.96948 | -68.18534 | 21Feb2021 | PQ558499 |  |
| LH | R | 001 | *L. flavipes* | *L. g. hexapetala* | Galarza | Corrientes | Argentina | -28.09045 | -56.70984 | 01Dec2021 | PQ558500 | PQ621634 |
| LH | R | 002 | *L. flavipes* | *L. g. hexapetala* | Galarza | Corrientes | Argentina | -28.09045 | -56.70984 | 01Dec2021 |  |  |
| MA | S | 001 | *L. flavipes* | *M. aquaticum* | Villa Regina | Río Negro | Argentina | -39.09821 | -67.07537 | 29Mar2022 |  |  |
| MA | S | 002 | *L. flavipes* | *M. aquaticum* | Villa Regina | Río Negro | Argentina | -39.09821 | -67.07537 | 29Mar2022 | PQ558513 |  |
| MA | T | 001 | *L. ciliersae* | *M. aquaticum* | Domingo Savio | Misiones | Argentina | -27.25561 | -55.29895 | 15Nov2022 | PQ558487 | PQ621630 |
| MA | T | 002 | *L. ciliersae* | *M. aquaticum* | Domingo Savio | Misiones | Argentina | -27.25561 | -55.29895 | 15Nov2022 | PQ558488 | PQ621631 |
| MA | T | 003 | *L. cilliersae* | *M. aquaticum* | Domingo Savio | Misiones | Argentina | -27.25561 | -55.29895 | 7Jan2024 | PQ556198 |  |
| MA | T | 004 | *L. cilliersae* | *M. aquaticum* | Domingo Savio | Misiones | Argentina | -27.25561 | -55.29895 | 7Jan2024 | PQ556199 |  |
| MA | T | 005 | *L. cilliersae* | *M. aquaticum* | Domingo Savio | Misiones | Argentina | -27.25561 | -55.29895 | 7Jan2024 | PQ558489 |  |
| MA | T | 006 | *L. cilliersae* | *M. aquaticum* | Domingo Savio | Misiones | Argentina | -27.25561 | -55.29895 | 7Jan2024 |  |  |
| MA | T | 007 | *L. cilliersae* | *M. aquaticum* | Domingo Savio | Misiones | Argentina | -27.25562 | -55.29896 | 7Jan2024 | PQ558490 |  |
| LM | U | 001 | *L. flavipes* | *L. p. montevidensis* | Bahia Blanca | Buenos Aires | Argentina | -38.69933 | -62.32927 | 28Feb2021 | PQ558505 |  |
| LM | U | 002 | *L. flavipes* | *L. p. montevidensis* | Bahia Blanca | Buenos Aires | Argentina | -38.69933 | -62.32927 | 28Feb2021 | PQ558506 |  |
| LH | V | 001 | *L. flavipes* | *L. g. hexapetala* | Laguna Brava | Corrientes | Argentina | -27.49034 | -58.71573 | 29May2022 | PQ558501 | PQ621635 |
| LH | V | 002 | *L. flavipes* | *L. g. hexapetala* | Laguna Brava | Corrientes | Argentina | -27.49034 | -58.71573 | 29May2022 | PQ558502 |  |
| LH | W | 001 | *L. flavipes* | *L. g. hexapetala* | Brazo Largo | Entre Ríos | Argentina | -33.86530 | -58.88294 | 05Feb2022 |  |  |
| LH | W | 002 | *L. flavipes* | *L. g. hexapetala* | Brazo Largo | Entre Ríos | Argentina | -33.86530 | -58.88294 | 05Feb2022 | PQ558503 | PQ621636 |

**S.3 Sequences obtained from public database GenBank.**

**Table S3.1**: Sequences obtained from GenBank

| **Taxon** | **Group** | **COI** | | **16S** | |
| --- | --- | --- | --- | --- | --- |
|  |  | **GenBank Code** | **COI** | **GenBank Code** | **16S** |
| Lysathia ludoviciana | ingroup | EU117150 | 764 (7 indels) |  |  |
| Lysathia ludoviciana | ingroup | EU117151 | 764 (8 indels) |  |  |
| Lysathia ludoviciana | ingroup | EU117152 | 764 (7 indels) |  |  |
| Lysathia ludoviciana | ingroup | EU117153 | 764 (7 indels) |  |  |
| Lysathia ludoviciana | ingroup | EU117154 | 764 (7 indels) |  |  |
| Lysathia ludoviciana | ingroup | EU1171551 | 764 (7 indels) |  |  |
| Lysathia ludoviciana | ingroup | EU117156 | 764 (6 indels) |  |  |
| Lysathia ludoviciana | ingroup | EU143722 | 764 (7 indels) |  |  |
| Lysathia sp | ingroup | EU117157 | 764 (7 indels) |  |  |
| Lysathia sp | ingroup | EU143717 | 764 (7 indels) |  |  |
| Altica ampelophaga | outgroup | KP763035 | 764 (7 indels) | KP763162 |  |
| Altica cirsicola | outgroup | DQ865064 | 764 (7 indels) | KC185980 | 387 (21 indels) |
| Altica cyanea | outgroup | KU697389 |  | KU697553 |  |
| Altica ericeti | outgroup | KP763068 | 764 (7 indels) | KP763192 | 432 (21 indels) |
| Altica fragariae | outgroup | KJ803204 | 764 (7 indels) |  |  |
| Altica impressicollis | outgroup | KF163284 | 764 (7 indels) |  |  |
| Altica oleracea | outgroup | KF163215 | 764 (7 indels) |  |  |
| Altica palustris | outgroup | KF163306 | 764 (7 indels) |  |  |
| Altica quercetorum | outgroup | KF163227 | 764 (7 indels) |  |  |
| Altica sp | outgroup | EU143714 | 764 (7 indels) |  |  |
| Altica tamaricis | outgroup | KF163274 | 764 (7 indels) |  |  |
| Altica viridicyanea | outgroup | JN903059 | 764 (7 indels) | FJ973794 | 451 (20 indels) |
| Diorhabda carinulata | outgroup | JQ782478 | 764 (7 indels) |  |  |
| Galerucinae sp | outgroup | LT160530 | 764 (7 indels) | FJ973875 | 451 (20 indels) |
| Longitarsus dorsalis | outgroup | KP763018 | 764 (7 indels) | KP763149 | 432 (25 indels) |
| Monolepta sp | outgroup | LT160545 | 764 (7 indels) | KC185951 | 387 (20 indels) |
| Podontia lutea | outgroup | FJ977985 | 764 (7 indels) |  |  |
| Psylliodes affinis | outgroup | KP763062 | 764 (7 indels) | KP763187 | 357 (21 indels) |
| Psylliodes chalcomerus | outgroup | HQ164766 | 764 (7 indels) |  |  |
| Systena blanda | outgroup | FJ977995 | 764 (7 indels) | FJ973874 | 450 (20 indels) |

**S.4 - Complete Phylogenetic Analysis Trees -**


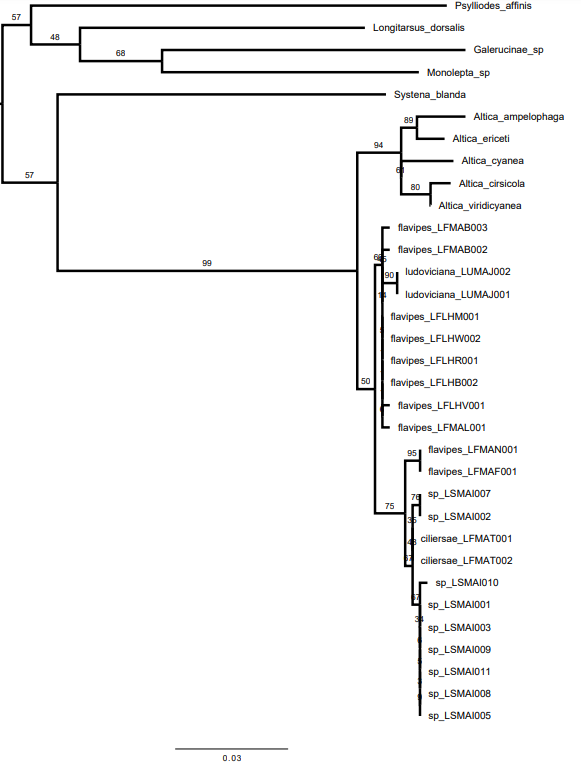


**Figure S4.1**: RAxML Phylogenetic Tree Based on the Ribosomal 16S Gene


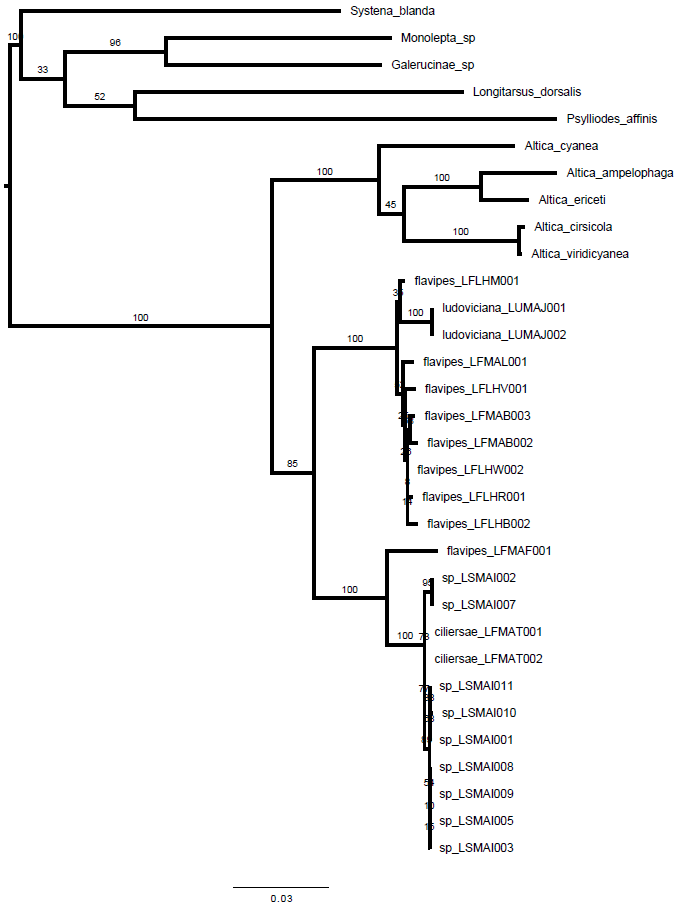


**Figure S4.2:** Phylogenetic tree (RAxML) based on mitochondrial COI and ribosomal 16S genes

**S.5 - Partial morphological description for *Lysathia* sp.1 (code MA_F_001) -**

Female. This species is distinct from the others in all cladograms and exhibits the following characteristics: It is a slender species, small size (Length = 3.45mm, maximum Width = 0.75mm), with violet colouration, differing in features of the antennae, legs, and female genitalia.

**Antennae:** The second antennomere is oblong, more than half the length of the third; the third appears slightly shorter than the fourth; antennomeres 4-6 are similar and the longest; antennomeres 7, 8, and 9 are thinner and longer than the preceding ones.
**Legs:** The hind femora are not as expanded as in L. flavipes and L. cilliersae, and the overall leg colour is light chestnut (or appears so), darker than in the other species studied.
**Genitalia:** The spermatheca has a cylindrical and elongated receptacle and pump; the receptacle is only slightly broader, and the pump is somewhat shorter, reaching just the base of the receptacle. The basal part of the spermathecal duct is thick and conical. The lamina of the sternite 8 is spatulate, with greater sclerotisation near the distal part of the lamina. The tignum is nearly three times the length of the lamina, slightly broadened at the base, quadrangular in shape. The coxites are short and divergent, with the baculi not reaching the sclerotised distal part of the coxites.
**Additional feature:** The proesternal process extends beyond the anterior coxal boundary.
